# Role of Hypoxia Inducible Factor-1α (HIF-1α) in Innate Defense against Uropathogenic *Escherichia coli* Infection

**DOI:** 10.1371/journal.ppat.1004818

**Published:** 2015-04-30

**Authors:** Ann E. Lin, Federico C. Beasley, Joshua Olson, Nadia Keller, Robert A. Shalwitz, Thomas J. Hannan, Scott J. Hultgren, Victor Nizet

**Affiliations:** 1 Division of Pediatric Pharmacology & Drug Discovery, University of California, San Diego, La Jolla, California, United States of America; 2 Aerpio Therapeutics, Cincinnati, Ohio, United States of America; 3 Department of Molecular Microbiology, Washington University School of Medicine, St. Louis, Missouri, United States of America; 4 Center for Women’s Infectious Disease Research, Washington University School of Medicine, St. Louis, Missouri, United States of America; 5 Skaggs School of Pharmacy and Pharmaceutical Sciences, University of California, San Diego, La Jolla, California, United States of America; 6 Rady Children’s Hospital, San Diego, California, United States of America; Northwestern University, Feinberg School of Medicine, UNITED STATES

## Abstract

Uropathogenic *E*. *coli* (UPEC) is the primary cause of urinary tract infections (UTI) affecting approximately 150 million people worldwide. Here, we revealed the importance of transcriptional regulator hypoxia-inducible factor-1 α subunit (HIF-1α) in innate defense against UPEC-mediated UTI. The effects of AKB-4924, a HIF-1α stabilizing agent, were studied using human uroepithelial cells (5637) and a murine UTI model. UPEC adherence and invasion were significantly reduced in 5637 cells when HIF-1α protein was allowed to accumulate. Uroepithelial cells treated with AKB-4924 also experienced reduced cell death and exfoliation upon UPEC challenge. *In vivo*, fewer UPEC were recovered from the urine, bladders and kidneys of mice treated transurethrally with AKB-4924, whereas increased bacteria were recovered from bladders of mice with a HIF-1α deletion. Bladders and kidneys of AKB-4924 treated mice developed less inflammation as evidenced by decreased pro-inflammatory cytokine release and neutrophil activity. AKB-4924 impairs infection in uroepithelial cells and bladders, and could be correlated with enhanced production of nitric oxide and antimicrobial peptides cathelicidin and β-defensin-2. We conclude that HIF-1α transcriptional regulation plays a key role in defense of the urinary tract against UPEC infection, and that pharmacological HIF-1α boosting could be explored further as an adjunctive therapy strategy for serious or recurrent UTI.

## Author Summary

Urinary tract infection (UTI), commonly caused by uropathogenic *E*.*coli* (UPEC), affects more than 150 million people worldwide, resulting in 14 million hospital visits per year and an estimated total cost of 6 billion dollars in direct health care. Due to the high prevalence of UTI and rapid emergence of antibiotic-resistant bacteria, new effective strategies to prevent and treat UTI are urgently needed. Here, we describe a global regulatory role of transcription factor hypoxia-inducible factor-1 (HIF-1) in innate antimicrobial defense against UPEC. HIF-1 stabilization reduces UPEC attachment to and invasion of uroepithelial cells, and protects bladders from UPEC-mediated cytotoxicity *in vivo*. In the murine UTI model, we found normal bladder HIF-1 expression is required for efficient UPEC clearance, since HIF-1-deficient mice suffer more severe infection than normal mice. Further studies showed that key elements of host protection provided by HIF-1 regulation are uroepithelial cell nitric oxide and antimicrobial peptide production. This study provides valuable insight into the importance of HIF-1 in supporting host immunity during UTI and its potential as a therapeutic target.

## Introduction

Urinary tract infection (UTI) is a very common and frequently recurrent bacterial disease accounting for approximately 8 million doctor visits per year, with an estimated total cost of $450 million annually in the United States [1,2]. More than 50% of women will experience at least one UTI during their lifetime, and about 30–40% of UTI recur within 6 months [3]. The invasive pathogen uropathogenic *E*. *coli* (UPEC) is a primary etiologic agent of UTI, causing severe bladder infection (cystitis) and acute kidney infections (pyelonephritis) [4,5]. To successfully establish infection, UPEC must first attach to superficial facet cells of bladder epithelium (uroepithelium), followed by invasion (entry) into the cytosolic milieu of these cells. UPEC colonization and invasion triggers an acute inflammatory response in the bladder epithelium leading to release of multiple pro-inflammatory cytokines including interleukin 6 (IL-6) and IL-1β, and chemokines such as IL-8 [6–10], and recruitment of neutrophils, which appear in the urine [11,12]. The inflammatory consequences of this early innate immune response can promote structural damage and cell death, including rapid shedding of the superficial uroepithelial cell layer lining the surface of the bladder lumen, which is a hallmark of UPEC infection [13,14]. Although pro-inflammatory activation is an important first line of defense against pathogens, an excessive response can promote chronic cystitis and acute or chronic pyelonephritis [15–18].

Hypoxia inducible factor-1α (HIF-1α) is a transcriptional regulator that orchestrates the cellular response to low oxygen stress. HIF-1α is degraded at normoxia through a prolyl-hydroxylase (PHD) and proteasome-dependent pathway, but under low oxygen (hypoxic) conditions translocates to the nucleus, where it activates expression of multiple gene targets including glucose transporters, enzymes of glycolysis, erythropoietin, and vascular endothelial growth factor [19]. An emerging literature has revealed significant intersection between the hypoxic and innate immune responses, and HIF-1α is now recognized to play key role in modulating innate immune cell function [20–22]. Mice with targeted deletion of HIF-1α in myeloid cells (neutrophils and macrophages) and skin or corneal keratinocytes are more susceptible to bacterial infection, with diminished antimicrobial peptide (AMP) and nitric oxide (NO) production and impaired microbicidal capacity demonstrable in the corresponding HIF-1α-deficient cells [23–25]. Conversely, pharmacological stabilizers of HIF-1α (PHD inhibitors) are in clinical development for treatment of anemia [26,27], and the potential for repositioning such drugs as “innate immune boosters” has been explored in animal models of bacterial infection. HIF-1α stabilizing agents increase the bactericidal capacity of phagocytic cells and epithelial cells [24,25,28,29], show therapeutic benefit in mouse models of *Staphylococcus aureus* skin infection [28,29], reduce intestinal tract inflammation and bacterial translocation in murine chemically-induced colitis [30], and support host defense during early stages of mycobacterial infection in zebrafish [31].

The dynamics of HIF-1α expression has been studied in the context of bladder cancer [32]; however, its role during bladder infections has not been explored. In this study, we couple *in vitro* and *in vivo* models of UTI to examine the role of HIF-1α in host defense of the urinary tract against UPEC, with an eye toward pharmacological HIF-1α boosting as a novel therapeutic approach in this highly prevalent and difficult to manage infectious disease condition.

## Results

### AKB-4924 stabilizes HIF-1α protein and reduces UPEC-mediated cytotoxicity and infection of human uroepithelial cells

**Fig 1:**
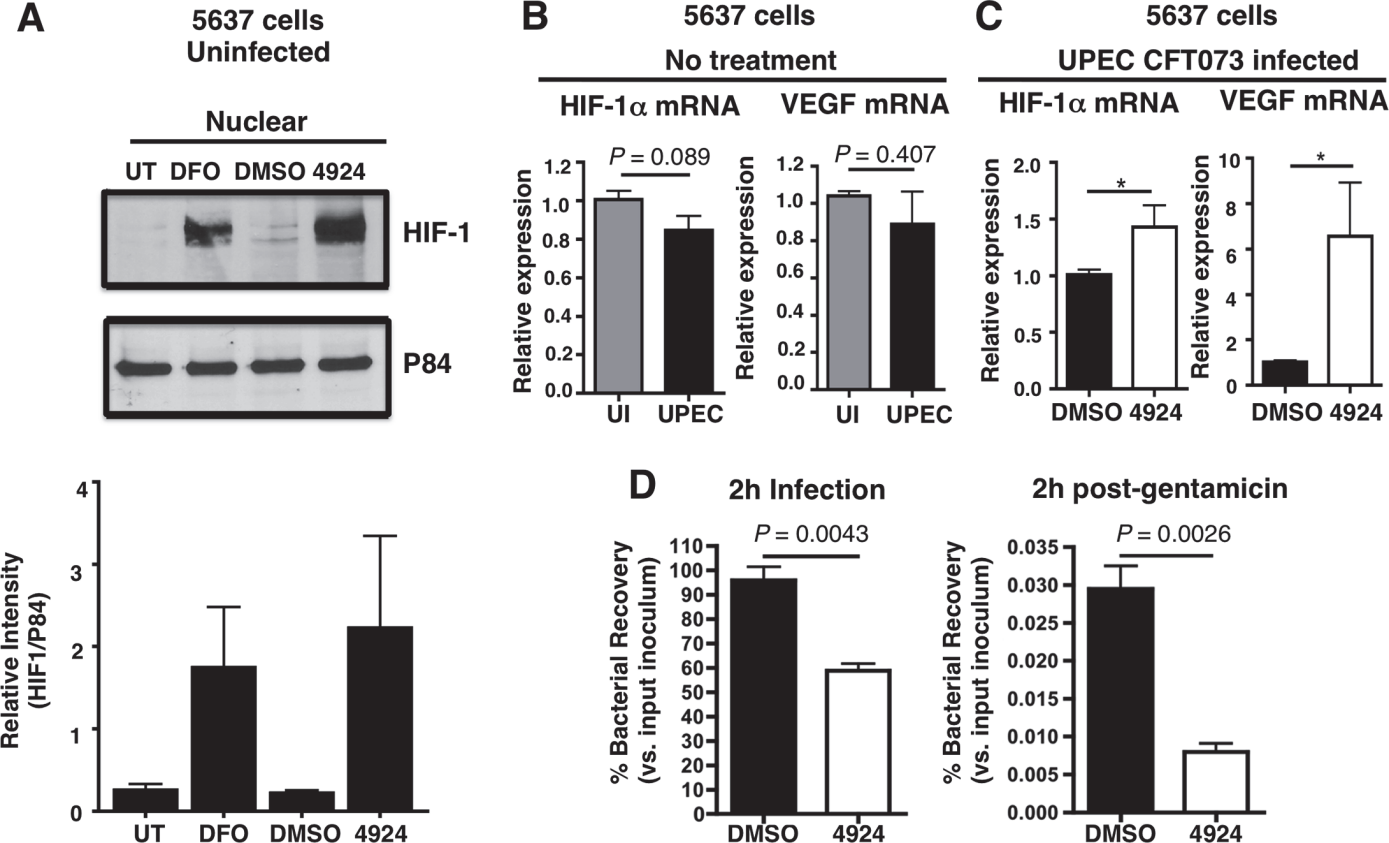
AKB-4924 stabilizes HIF-1α protein and reduces UPEC-mediated infection of cultured human uroepithelial cells. (**A**) Western blot illustrating expression of HIF-1α (120 kDa) in the nuclear fraction of uninfected human uroepithelial cell line 5637 left untreated (UT), or treated with DMSO (negative control), DFO (desferrioxamine mesylate; positive control) or AKB-4924 for 2 h. Nuclear matrix protein P84 was used as loading control. Bar graph shows relative intensity of protein bands from repeated experiments (n = 2). (**B**) Real-time qPCR showing relative HIF-1α and VEGF mRNA expression in uninfected (UI) or UPEC CFT073 infected (2 h) 5637 cells without treatment, or (**C**) DMSO (mock treatment and 4924 pre-treated cells followed by UPEC infection (n = 10) (**D**) Bacterial counts from 2 h DMSO or AKB-4924 treated 5637 cells followed by 2 h infection with UPEC CFT073 to measure total bacteria, or 2 h infection with additional 2 h gentamicin (100 μg/mL) treatment to measure intracellular bacteria (n = 3 per group). Shown as mean +/- S.E.M., *P < 0.05, Student’s unpaired t-test. Results are pooled from three independent experiments.

**Fig 2:**
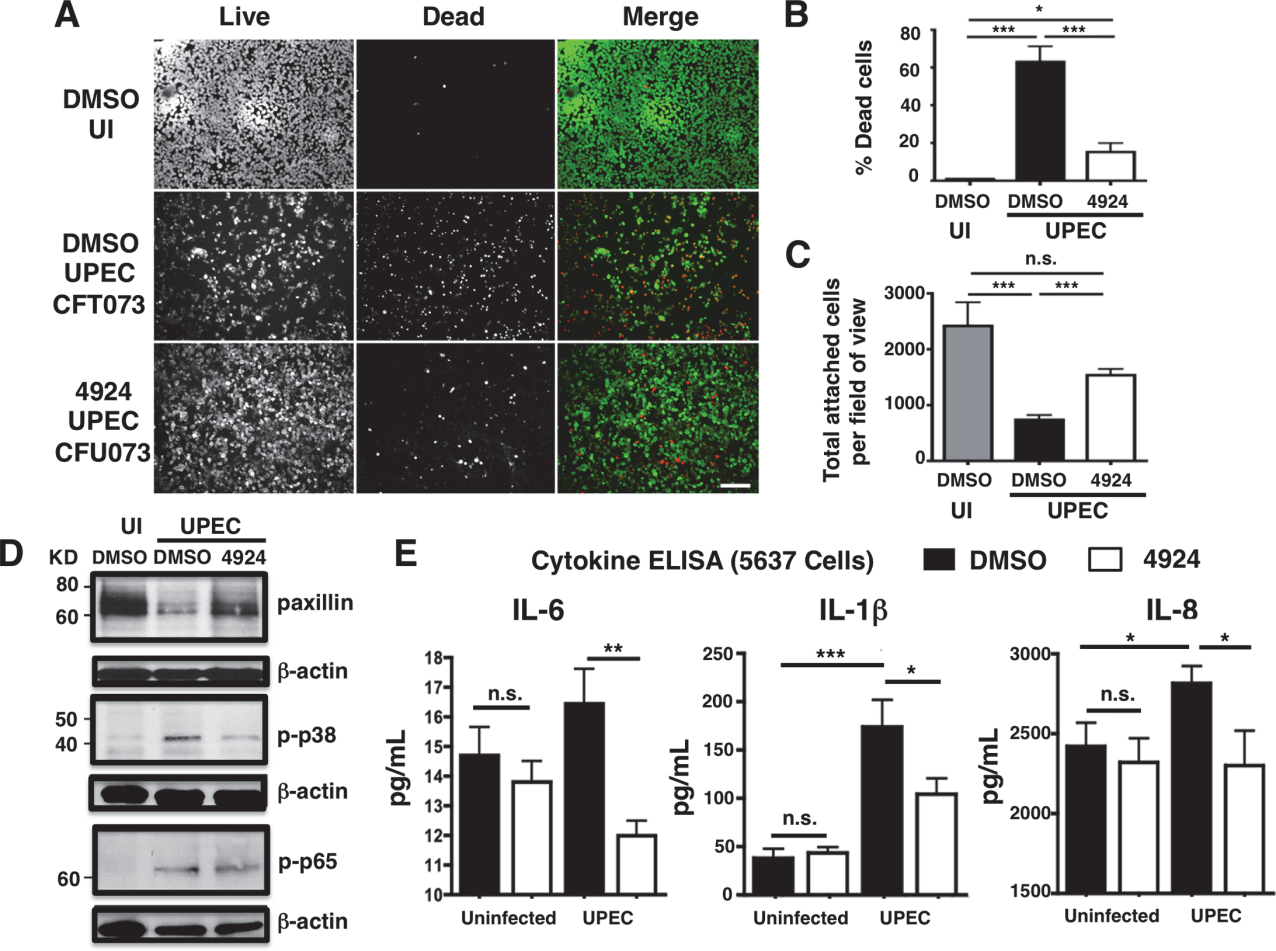
AKB-4924 reduces UPEC-mediated cytotoxicity and inflammation in human uroepithelial cells. 5637 Cells were infected with UPEC CFT073 for 2h at MOI ~20. (**A**) Live/dead cell staining illustrates viable (green) or dead (red) cells. Scale bar = 100 μm. (**B**) Percentage death in uninfected (UI), DMSO and AKB-4924 pre-treated 5637 cells (n > 7). (**C**) Total attached cells per field of view. Counts were made from multiple random fields of view (10x objective, n > 7) from independent samples (n = 3). Error bar = S.E.M, ***P < 0.001, **P < 0.01, *P < 0.05 by student’s two-tailed unpaired t-test. (**D**) Western blots of paxillin, phospho-p38 and phospho-p65 expression of uninfected (UI), UPEC-infected 5637 cells with prior exposure of DMSO or AKB-4924 (n = 3 per group). Data represent one of two independent experiments. (**E**) ELISA detection of pro-inflammatory cytokine proteins IL-6, IL-1β and IL-8 released by DMSO or AKB-4924 pre-treated 5637 cells 2 h post- infection (n = 4 per group). Results are pooled from 3 independent experiments.

AKB-4924 (Aerpio Therapeutics) is a prolyl-hydroxylase inhibitor drug candidate that preferentially stabilizes HIF-1α and increases phagocyte killing of *S*. *aureus in vitro* and in a murine skin infection model [29]. We found that treatment with 20 μM AKB-4924 for 2 h significantly increased HIF-1α protein abundance in healthy human uroepithelial cell line 5637 (ATCC HTB-9), comparable to the effect of the hallmark HIF-1α agonist desferrioxamine mesylate (DFO) (**Fig 1A**). This result confirms that AKB-4924 stabilizes HIF-1α to prevent its degradation; resulting in an increase in HIF-1α protein. HIF-1α expression in human 5637 cells is not significantly altered during UPEC CFT073 infection (**Fig 1B**), but becomes upregulated upon AKB-4924 treatment (**Fig 1C**). To corroborate this result, we examined the transcription level of the gene encoding vascular endothelial growth factor (VEGF), a peptide cytokine classically induced by HIF-1α at the transcriptional level [25,33]. AKB-4924 treatment increased VEGF mRNA by approximately 6-fold (**Fig 1C**). Thus, to examine how AKB-4924-mediated HIF-1α upregulation influenced UPEC survival during uroepitheilal cell infection, we recovered bacterial colony forming units (CFU) 2 h post-infection (total bacterial survival), or after a subsequent 2 h of gentamicin treatment, a standard method frequently used to assess intracellular bacterial survival due to its poor ability to penetrate mammalian cell membranes [34–38]. At 100 μg/mL, gentamicin effectively kills extracellular UPEC, as no detectable colonies were recovered from the media containing gentamicin after 2 h treatment [39]. We observed a marked reduction (~40%) in UPEC CFU recovery from 5637 cells pre-treated with AKB-4924 at both time points, compared to mock (DMSO-treated cells (**Fig 1C**). Likewise, we found infection of UPEC UTI89, a hyper-invasive strain, was also significantly attenuated in AKB-4924 treated uroepithelial cells (**S1 Fig**). UPEC are known to trigger rapid death and extensive exfoliation of the uroepithelial layer of the bladder [40,41]. Through Live/Dead cell staining (**Fig 2A**), we documented a clear reduction (~40%) in UPEC-induced cell death and detachment in AKB-4924 pre-treated cells (**Fig 2B and 2C**). UPEC induce degradation of paxillin, a focal adhesion molecule important for cell attachment [42]. Western blot revealed that paxillin levels were partially restored in AKB-4924 treated bladder cells infected with UPEC (**Fig 2D**), likely contributing to preservation of cell integrity and surface attachment. Treatment of AKB-4924 did not alter paxillin level in uninfected cells **(S2B Fig**). Similar to our *in vitro* findings, mice pre-treated with AKB-4924 showed less paxillin degradation compared to mock treated animals after UPEC CFT073 infection (**S2A Fig**).

UPEC cytotoxicity to uroepithelial cells is mediated through the mitogen-activated protein kinase (MAPK)-dependent pathway, particularly via phospho-p65 and phospho-p38 MAPK activation [39,43–45]. While AKB-4924 treatment did not reduce UPEC-mediated p65 phosphorylation, it partially blocked UPEC-mediated p38 MAPK activation (**Fig 2D**). Neither phospho-p65 nor phospho-p38 was altered by AKB-4924 in uninfected cells (**S2B Fig**). Our *in vivo* study also revealed that AKB-4924 treated bladders with UPEC infection had diminished p38 activation (**S2A Fig**). The p38 MAPK plays a key role in initiating cell death pathways and triggering multiple downstream pro-inflammatory cytokine responses in epithelial cells [46,47]. UPEC-infected cells released significantly less pro-inflammatory cytokines known to be regulated by p38 MAPK, including IL-6, IL-1x and IL-8 [47,48], in the presence of AKB-4924 (**Fig 2E**). In contrast, AKB-4924 did not alter the cytokine production profile in uninfected cells.

### Pharmacological HIF-1α stabilization restricts UPEC urinary tract colonization in mice

**Fig 3:**
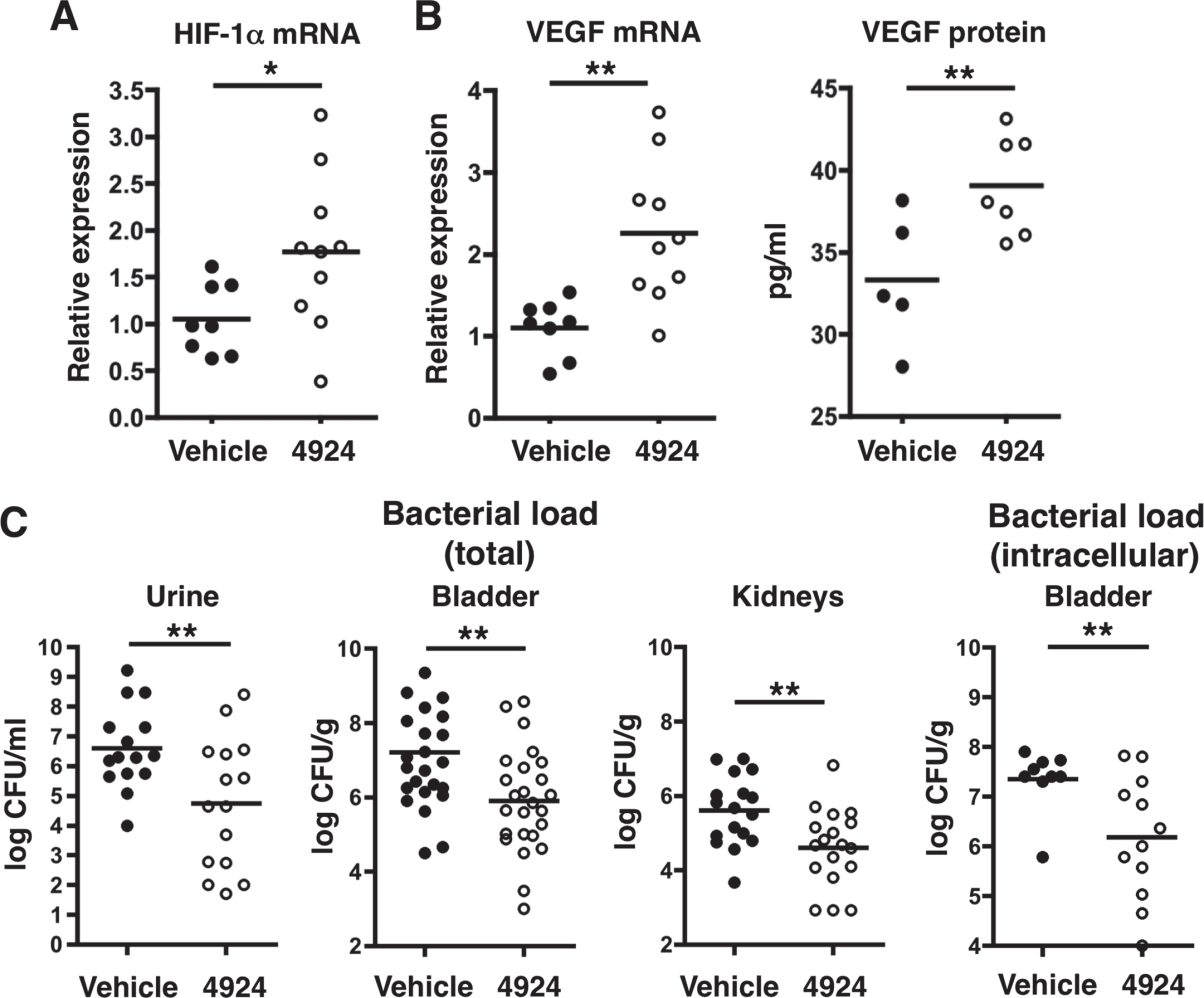
Pretreatment with AKB-4924 impairs UPEC urinary tract colonization in C57BL/6 mice. (**A**) Bladders from female C57BL/6 mice treated with 1 h vehicle, or 0.2 mg/mL AKB-4924, were recovered after 18–24 h UPEC CFT073 infection. Real-time qPCR shows increased Hif-1 mRNA in AKB-4924 treated animals. (**B**) Real-time qPCR and ELISA show increased VEGF mRNA and protein expression in AKB-4924 treated group. Data are generated from two independent repeats (n > 5 per experiment). (**C**) UPEC recoveries from urine (n = 15), bladder (n = 25) and both kidneys (n = 18) of mice that received 1 h of 4924 pre-treatment were significantly decreased compared to vehicle treated mice. Data is presented as mean +/- SEM, generated from 3–4 independent experiments, **P < 0.01. *Ex vivo* gentamicin protection assay revealed intracellular bacterial CFU from mice 18 h post-infection (n = 10,12)

To examine the potential utility of AKB-4924 in preventing UPEC infection *in vivo*, we used a well-described murine UTI model [49]. Each mouse received 0.2 mg of AKB-4924 via transurethral injection for 1 h prior to initiation of an 18–24 h UPEC infection. AKB-4924 treated animals had an approximately 2-fold increase in HIF-1α mRNA recovered from their bladders upon infection (**Fig 3A**). In contrast, UPEC infected animals without AKB-4924 treatment showed no changes in HIF-1α mRNA levels (**S3A Fig**). AKB-4924 also elevated productions of VEGF mRNA and protein in the bladders at different infection time points (**Fig 3B and S4A Fig**). Pharmacological HIF-1α activation remarkably decreased bacterial burden in the urine, bladder and kidneys compared to vehicle treated mice 18–24 h post-infection (**Fig 3C**). Almost 50% of mice assayed had urine titers under 10^4^ CFU/mL, which is a commonly applied cut-off for resolution of experimental bacteriuria in mice [18,50]. Thus AKB-4924 protects against development of localized UPEC bladder infection. Through a time-course analysis, AKB-4924 was shown to impair UPEC colonization in the bladder from 4 h to 8 h post-infection. Mock-treated mice had significantly higher bacterial burdens at 8 h compared to 4 h, whereas the bacterial burden in 4924-treated mice remained similar in this interval (**S4C Fig**). Interestingly, many mice challenged with UPEC also displayed high levels of CFU recovery in kidneys 18–24 h post-infection; AKB-4924 treatment reduced bacterial recovery from kidneys (**Fig 3C**), indicating that AKB-4924 impedes ascending UTI. One of the key events during UPEC infection is intracellular invasion of uroepithelial cells, upon which time the bacteria rapidly develop into intracellular bacterial communities (IBCs) [12]. To determine whether AKB-4924 prevents UPEC invasion into the bladder tissue, we treated bladders *ex vivo* with gentamicin to kill extracellular UPEC to assess level of intracellular bacteria within the tissue. Consistent with the total CFU recovered from the bladder and the *in vitro* gentamicin protection assay (**Fig 1B**), we found significantly less intracellular (gentamicin-protected) bacteria in the bladder of mice prophylactically treated with AKB-4924 at 18 h post-infection (**Fig 3C**). As a control, we excluded the possibility of a direct bactericidal effect of AKB-4924 on UPEC, as the drug did not influence UPEC growth in mouse urine or tissue culture media (RPMI supplemented with fetal bovine serum) over 20 h incubation at 37°C (**S3B Fig**). In addition to growth, we also tested whether AKB-4924 has any direct impact on UPEC fitness or flagella-driven motility. Assessed by a swimming motility assay using soft agar (0.25% LB agar), AKB-4924 did not alter UPEC motility through 18 h of growth (**S3C Fig**).

**Fig 4:**
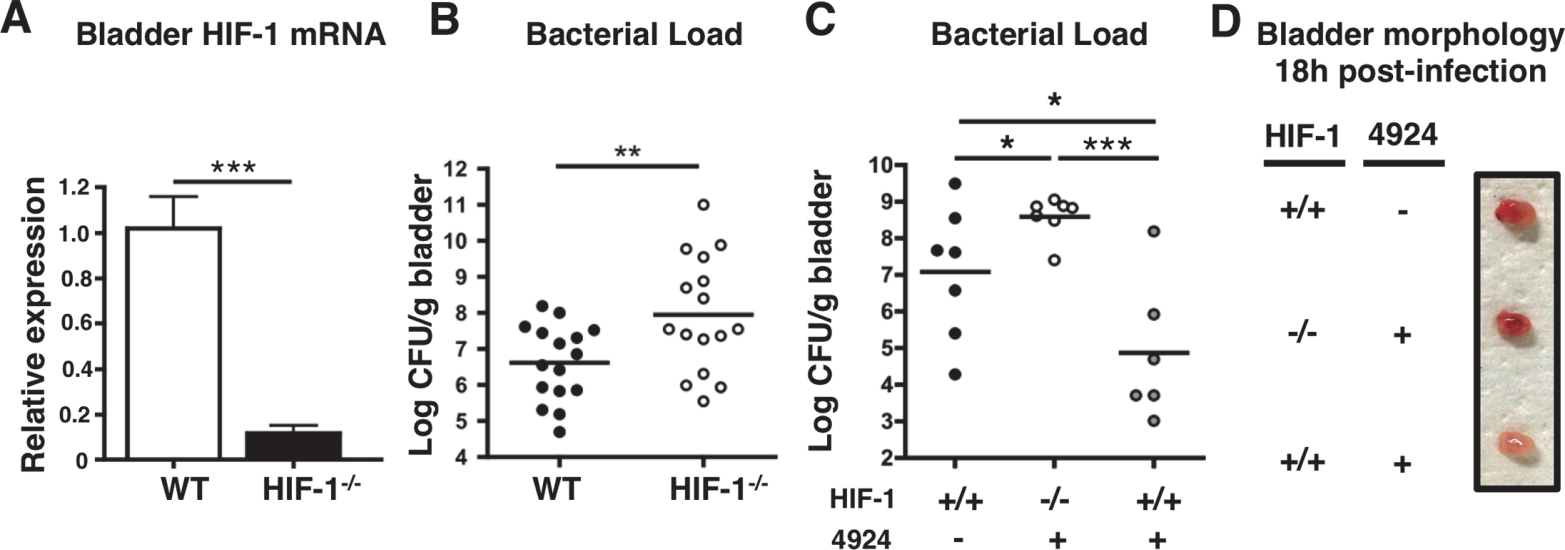
Mice lacking HIF-1 in their bladder epithelium are more susceptible to UPEC urinary tract colonization. (**A**) Level of Hif-1 transcripts in bladders of HIF-1^-/-^ (*Hif1αflox/flox*/K14-Cre^+^) mice compared to wild-type mice (n = 3). (**B**) WT or HIF-1^-/-^ mice were infected with UPEC CFT073 and bladders were harvested 24 h post-infection for CFU enumeration (n = 16). (**C**) WT or HIF-1^-/-^ mice were pre-treated with 1 h of 0.2mg of AKB-4924 and infected with UPEC. Bladders were harvested 18 h post-infection for CFU enumeration (n = 7). (**D**) Images of representative UPEC infected bladders from littermates (n = 3–4 per group) with or without prior AKB-4924 treatment. Error bar = S.E.M ***P < 0.001, **P < 0.01, *P < 0.05, Student’s two-tailed unpaired t-test. Results are mean values from 2 or more independent experiments.

A genetic approach was used to verify the importance of HIF-1α in uroepithelial defense UPEC infection. In mice with a Cre recombinase-mediated, keratinocyte-specific inactivation of HIF-1α, (*Hif1α* ^*flox/flox*^/K14-Cre^+^, hereafter known as HIF-1^-/-^) [24], we confirmed an 80% reduction in bladder HIF-1α mRNA by real-time quantitative PCR (qPCR) compared to wild-type (WT) littermate controls (**Fig 4A**). Upon UPEC challenge, these HIF-1^-/-^ mice were more susceptible to bladder infection than WT mice (**Fig 4B)**. AKB-4924 pre-treatment did not alter infection severity or bacterial counts in the bladders of HIF-1^-/-^ mice, in contrast to the protective effects observed in AKB-4924 treated WT mice (**Fig 4C**). This confirms that AKB-4924-mediated protection against UPEC infections requires HIF-1α, and emphasizes the importance of HIF-1α stabilization in attenuating UTI. Gross morphology showed markedly reduced hemorrhagic inflammation in WT mice treated with AKB-4924 compared to untreated WT mice or AKB-4924 treated HIF-1^-/-^ mice (**Fig 4D**). These results confirm that the AKB-4924 therapeutic effect occurs via HIF-1 boosting and not an off-target activity, and provide further evidence that HIF-1α plays a key role in mitigating the development of UTI.

### HIF-1α stabilization reduces UPEC-mediated inflammatory damage to bladder epithelium

**Fig 5:**
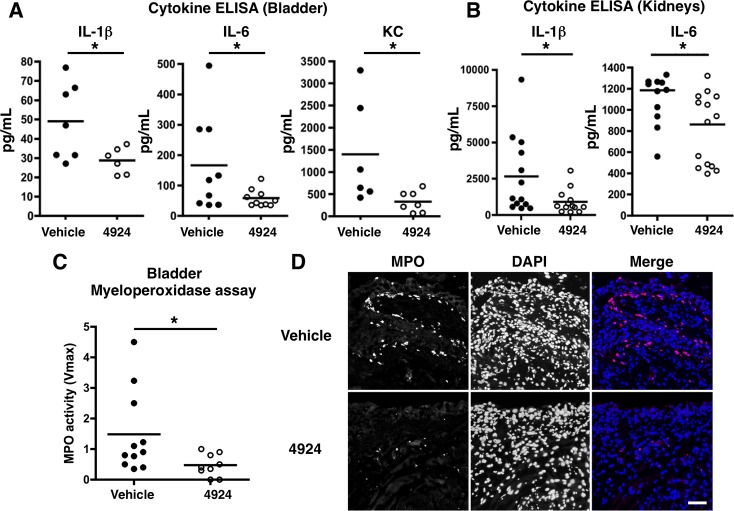
Reduced UPEC-mediated inflammatory damage to bladder epithelium with AKB-4924 pretreatment. (**A, B**) ELISA analysis shows pro-inflammatory cytokines IL-6, IL-1β or KC in the bladders or kidneys are reduced in AKB-4924 treated mice 18–24 h post UPEC CFT073 infection. Results are compiled from more than two independent experiments (n > 3 per group). (**C**) Myeloperoxidase (MPO) assay of bladder supernatants (n = 9–11) indicates significant decrease in MPO activity in AKB-4924 pre-treated bladders 18h post UPEC CFT073 infection; Error bar = S.E.M *P < 0.05, Student’s two-tailed unpaired t-test. (**D**) Representative immunolocalization images of bladder MPO (red) and nuclei (DAPI, blue) of 18 h UTI89-infected mice with or without 4924 pre-treatment (n = 3–4 per group). Scale bar = 50 μm.

To examine the inflammatory profile of UPEC-infected bladders, we measured the levels of secreted IL-1β, IL-6 and KC (keratinocyte-derived chemokine, a murine ortholog of the human neutrophil chemokine CXCL1) in mouse bladder homogenate following 1 h pre-treatment with AKB-4924 or vehicle control prior to infectious challenge. Consistent with our *in vitro* data (**Fig 1B and Fig 2E**), we observed a decrease in pro-inflammatory cytokine production in AKB-4924 pre-treated bladders (**Fig 5A**). Reduced IL-1β and IL-6 levels were also detected in kidneys of AKB-4924 treated mice (**Fig 5B**). Concomitant to these reduced cytokine levels, we observed a significant decrease in myeloperoxidase (MPO) activity, a marker of neutrophil recruitment [51], in AKB-4924 treated bladders compared to those treated with vehicle control (**Fig 5C**). Histology section from infected bladders with vehicle treatment displayed hyperplasia and edema, characterized by increases in crypt length within the uroepithelial lining; similar observations were previously made in other UPEC infected murine bladders [13,14,52,53]. In contrast, AKB-4924 treated mice exhibited decreased hyperplasia in the bladder epithelial layer (**S5 Fig**). We also noted a decrease in bacterial population in the luminal region of the AKB-4924 treated mice (**S5 Fig**). These observations reflect our previous cytokine measurement studies. As UPEC CFT073 caused only mild to moderate bladder inflammation, we repeated infection using the hyper-virulent UPEC strain UTI89 to trigger higher grade inflammation and further analyze the effects of AKB-4924 treatment. In the hyper-virulent UTI89 infection, MPO localization was monitored by immunofluorescent microscopy using a labeled anti-MPO antibody, revealing a significant reduction in MPO localization in the uroepithelial lining of AKB-4924 treated bladders (**Fig 5D).** MPO expression is nearly undetectable level in uninfected cells (**S6B Fig**), confirming the elevated level of MPO expression is not a pre-existing inflammatory condition but occurs specifically in response to UPEC infection. Together, these results indicate that HIF-1α stabilization via AKB-4924 treatment significantly dampens UPEC-mediated inflammatory responses to the bladder.

### HIF-1α stabilization enhances production of nitric oxide and host defense peptides during UPEC infection

**Fig 6:**
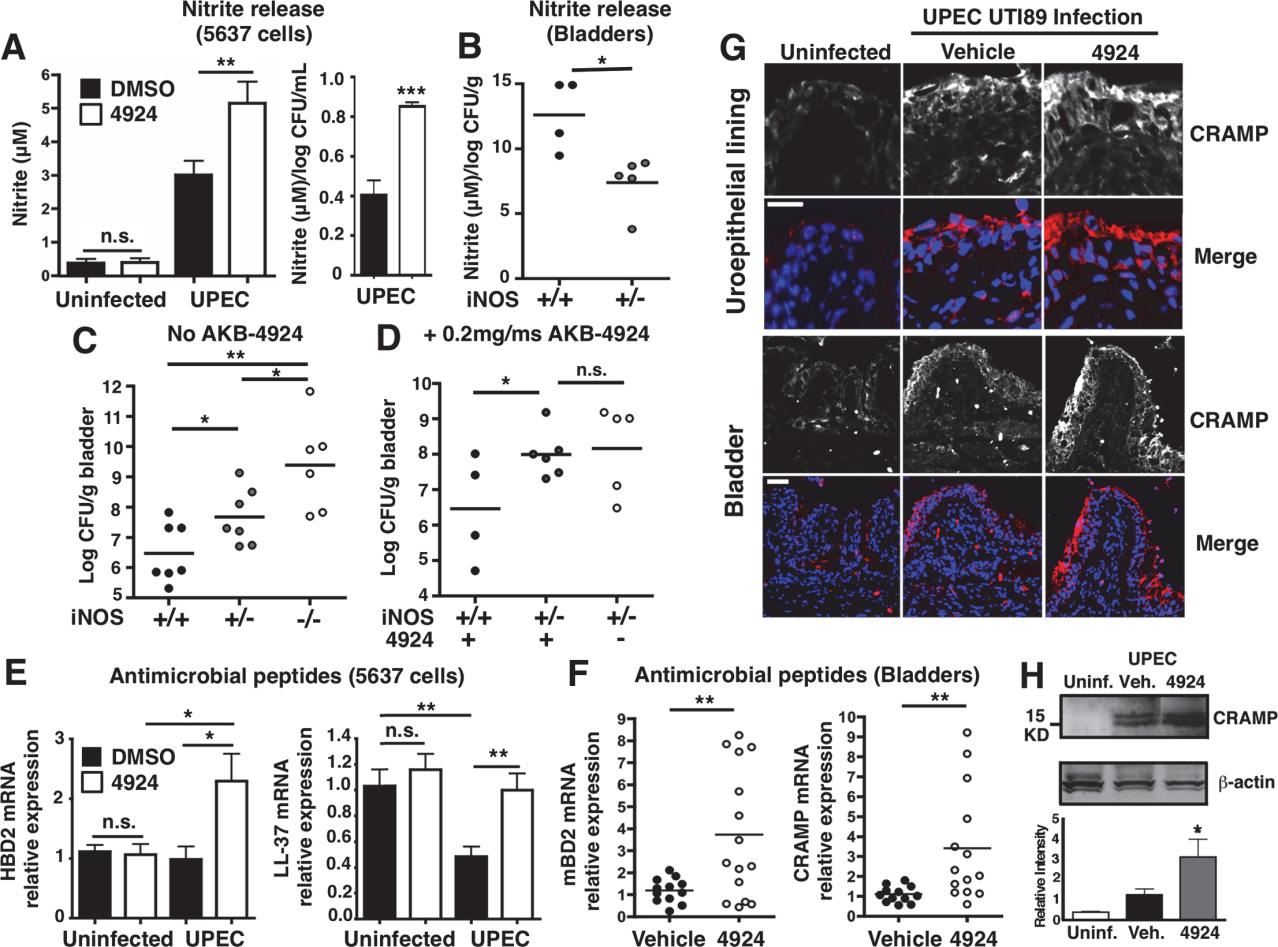
AKB-4924 pretreatment enhances production of nitric oxide and host defense peptides during UPEC infection. (**A**) Nitrite production from AKB-4924 treated 5637 cells was significantly higher than from DMSO treated cells 2 h post-infection. (Uninfected, n = 6; UPEC CFT073 infected, n = 12). Left panel: Absolute nitrite production (μM). Right panel: Relative nitrite productions per log CFU per mL (n = 6). (**B**) Nitrite released from WT (iNOS +/+) and heterozygous (iNOS +/-) per log CFU per bladder (gram) (n = 4–5) (**C**) WT (iNOS +/+), iNOS+/- and iNOS-/- C57BL/6 mice were infected with UPEC CFT073 for 18–24h before bladders were harvested for CFU enumeration (n = 7, 6). (**D**) WT and iNOS +/- C57BL/6 mice were pre-treated with 0.2 mg AKB-4924 or ωehicle for 1 h followed by infection with UPEC CFT073 (n = 4–6). (**E**) Human AMP β-defensin 2 (hBD2) (n = 8) and cathelicidin LL-37 (n = 7–10) mRNA level in UPEC CFT073 infected 5637 cells. Results were normalized to β-actin. (**F**) Real-time qPCR shows murine AMP βdefensin 2 (mβD2) and CRAMP mRNA level in bladders (n = 10–12). Error bar = S.E.M **P < 0.01, *P < 0.05, Student’s two-tailed unpaired t-test. (**G**) Representative images illustrating the distribution of murine cathelicidin (red) and nuclei (DAPI, blue) in the mucosa of UTI89 infected bladders with vehicle or AKB-4924 pre-treatment (n = 6). Upper panel scale bar = 50 μm. Bottom panel: higher magnitude focused on the uroepithelial cell layer of bladder. Scale bar = 25 μm. (**H**) Representative western blot of mouse bladder left uninfected (n = 2) or infected overnight with UPEC CFT073 (n = 3). Infected animals were pre-treated with vehicle or AKB-4924 for 2 h. Relative level of cathelicidin level was measured using Image J and normalized to β-actin from 2 independent western blot experiments (2–3 different animals in each western blot).

Since AKB-4924 does not display direct bactericidal activity against UPEC (**S3B Fig**), we hypothesized that the drug induced production of endogenous antimicrobial compounds by bladder epithelial cells to promote bacterial clearance. Patients with bladder infections are known to have increased inducible nitric oxide synthase (iNOS) and its product nitric oxide (NO), which can exert antimicrobial activity against susceptible pathogens [10]. Because HIF-1α is a transcriptional activator of iNOS [25,31,54], we assessed the functional output of iNOS activity by measuring levels of nitrite (NO_2_-), a stable NO oxidation product. Like previous studies that showed significant nitrite production in urinary pathogenic *E*. *coli* infected mouse bladders [55,56], we found UPEC also significantly induces nitrite production in human uroepithelial cells 5637 (**Fig 6A, left panel**). Nitrite induction was further augmented by AKB-4924 pre-incubation, but the drug did not produce an observable increase in nitrite levels in uninfected cells (**Fig 6A, left panel)**. UPEC can reduce nitrate to nitrite and also derive nitrite from iNOS-mediated NO production. We normalized the nitrite production level to total CFU per well (1 mL), highlighting that AKB-4924 double nitrite release from 5637 cells during infection (**Fig 6A, right panel)**. Increased nitrite production in AKB-4924 treated cells correlated to increased iNOS transcription (**S7A Fig**). To further analyze NO protection in UTI, we performed infection in a set of WT (iNOS +/+), iNOS heterozygous (iNOS +/-) and knockout (iNOS-/-) mice. iNOS knockout mice were significantly more susceptible to UPEC infection than either WT or iNOS heterozygous mice (**Fig 6C**). The increased bacterial load in iNOS +/- bladders corresponded with lower nitrite levels in these mice (**Fig 6B**), indicating NO contributes to effective bacterial clearance in the bladder. When WT and iNOS +/- mice were treated with AKB-4924 prior to UPEC CFT073 infection, we found that lacking a fully functional host iNOS system, the protective effect of AKB-4924 was significantly diminished; moreover, the effect of AKB-4924 was abolished in iNOS +/- mice (**Fig 6D**). Overall, our results demonstrate that the protection generated by HIF-1 boosting via AKB-4924 is partially dependent on NO production.

HIF-1α transcriptionally upregulates the expression of the cationic antimicrobial peptides cathelicidin (human = LL-37, mouse = mCRAMP) and/or β-defensin in neutrophils [25,29], keratinocytes [24] or cornea [23] to impede various pathogens including GAS, MRSA and *P*. *aeruginosa*. Uroepithelial cells secrete abundant quantities of peptides cathelicidin and β-defensin 2 in response to UPEC infections [57–59]. Cathelicidin has been shown to be important for UPEC clearance, since mCRAMP-deficient mice suffer significantly higher bacterial burdens during UTI [58]. Likewise, bladder epithelial cells that lack human β-defensin 2 (hBD2) are more susceptible to *E*. *coli* infection [60]. Based on these studies, we examined whether the reduced bacterial burden in AKB-4924-treated epithelial cells and bladders could be correlated with an increase in antimicrobial peptide production. Real-time qPCR revealed hBD2 was transcriptionally upregulated by two-fold in 5637 uroepithelial cells pre-exposed to AKB-4924, whereas UPEC infection alone did not alter hBD2 mRNA levels (**Fig 6E**). Interestingly, we found UPEC significantly suppressed LL-37 transcription in 5637 cells 2h post-infection, which mirrored results from Chromek and colleagues, where they demonstrated UPEC induced a rapid decrease in LL-37 transcript in uroepithelial cells after more than 2 h infection [58]. Remarkably, AKB-4924 restored LL-37 mRNA back to the pre-infection levels in cells 2 h post UPEC infection, a 2-fold increase compared to infected and vehicle-treated cells (**Fig 6E, right panel**). In correlation with these *in vitro* data, we found mRNA expression of mBD2 and mouse cathelicidin-related antimicrobial peptide (mCRAMP) was significantly augmented in AKB-4924 treated mice compared to vehicle-treated mice 18 h post-infection by approximately 4-fold (**Fig 6F**). Immunofluorescence studies in UPEC UTI89-infected mice revealed a clear increase in cathelicidin protein distribution in the bladder uroepithelial layer compared to uninfected mice (**Fig 6G**). A slight increase in cathelicidin expression was observed deeper into the bladder epithelium with AKB-4924 pre-treatment (**Fig 6G**). A similar increase in cathelicidin distribution was illustrated in a previous study in which vitamin D treatment was used to stimulate bladder expression of the peptide [57]. We confirmed our immunohistopathology results by western blot and densitometry, which showed that AKB-4924 pre-treatment increased cathelicidin protein levels by approximately 1.5-fold compared to vehicle controlled in UPEC CFT073 infected bladders (**Fig 6H**). This result is consistent with our prior study showing reduced cathelicidin expression in HIF-1^-/-^ mouse skin keratinocytes [24], emphasizing the key role of HIF-1α in regulating cathelicidin production. When we treated UPEC with filter-sterilized supernatants prepared from uroepithelial cells infected with UPEC, significantly fewer bacteria were recovered from supernatants from 4924-treated cells vs. controls (**S7B Fig**). Since AKB-4924 is not bactericidal (**S3B Fig**), HIF-1α stabilization increases the release of an antimicrobial factor from uroepithelial cells that contributes to bacterial killing.

### Delayed pharmacological HIF-1α stabilization ameliorates UPEC UTI in the mouse model

**Fig 7:**
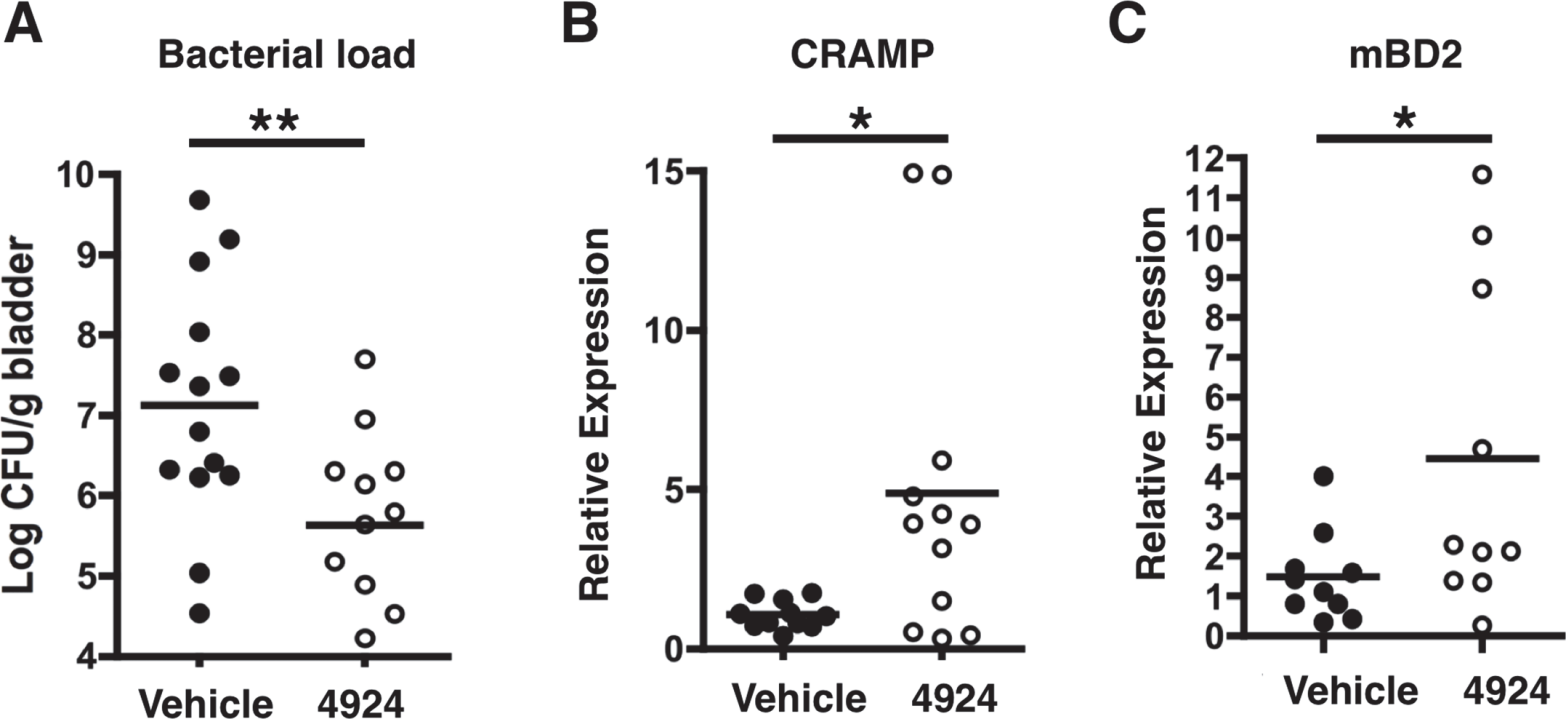
AKB-4924 attenuates urinary tract infection. (**A**) Bladders isolated from female C57BL/6 mice infected with UPEC CFT073 for 6 h received localized treatment with 0.2 mg of AKB-4924 via transurethral injection for 16 h. Less bacteria were recovered from infected bladders treated with AKB-4924 compared to vehicle. Real-time qPCR shows elevated level of (**B**) murine AMP CRAMP and (**C**) mBD2 mRNA (n >11) in AKB-4924 treated animals. Error bar = S.E.M, **P < 0.01, *P < 0.05, Student’s unpaired two-tailed t-test. Results are compiled from 2 independent experiments.

Having demonstrated that AKB-4924 pretreatment suppresses the establishment of UPEC infections *in vivo*, we assessed the value of AKB-4924 as a potential therapeutic agent for animals with established UPEC infection. Mice were first infected with UPEC and then 6 h later administered with 0.2 mg AKB-4924 transurethrally; this time point was selected based on previous studies indicating that it corresponded to the point of highest bacterial recovery [49], at which time the bacteria have attached and invaded epithelium to form early IBCs [61]. Mice treated with AKB-4924 showed more than a 10-fold reduction in UPEC colonization of the bladder (**Fig 7A**); a finding that coincided with an increase in CRAMP and hBD2 mRNA levels (**Fig 7B and 7C**). This finding suggests that AKB-4924 treatment can impede UPEC even in the face of established bacterial invasion and formation of early IBC in the bladder epithelium. Together, our combined data indicate that by stabilizing HIF-1α, AKB-4924 enhances production of antimicrobial effectors in both prophylactic and therapeutic settings, promoting bacterial clearance, and suggesting HIF-1α boosting as a potential adjunctive therapeutic strategy in UTI management.

## Discussion

Upon microbial infection, HIF-1α stabilization is part of the general myeloid cell innate immune response to elicit host protection [21,22,62,63]. HIF-1 is an oxygen-inducible master transcriptional activator that regulates several targets important for innate immune functions. Low oxygen levels inhibit ubiquitylation-mediated degradation of HIF-1, resulting in activation of HIF-1 transcriptional response. HIF-1 transcription is also regulated through NF-κB signaling [64]. Previous studies have suggested that patients suffering from UPEC display lower oxygen levels in their bladders [65,66], a phenomenon that could in theory further stabilize HIF-1. Nevertheless, we demonstrated that HIF-1α post-transcriptional regulation plays a key role in protecting against UPEC infection and injury to bladder epithelium *in vitro* and *in vivo*. AKB-4924 effectively increases HIF-1α stability in cultured human uroepithelial cell line 5637, paralleling studies which showed the drug can induce HIF-1 expression in murine fibroblasts, human monocytes [29] and intestinal epithelial cells [30]. AKB-4924 increases HIF-1α expression in UPEC-infected human uroepithelial cells *in vitro* and murine bladders *in vivo*, which may be potentiated by a positive feedback loop since HIF-1α positively auto-regulates its own expression by binding to the HIF-1α promoter [67]. In addition to HIF-1α, we found that AKB-4924 also stabilizes HIF-2α (**S2C Fig**). During AKB-4924 treatment (stabilizing both HIF-1 isoforms), HIF-1-/- animals experienced a significantly higher bacterial burden than WT animals, suggesting that HIF-1 plays the predominant role in regulating UPEC clearance.

Exposing human uroepithelial cells or mouse bladders to AKB-4924 prior to infection significantly blocked bacterial attachment and invasion, and dampened the host pro-inflammatory response and p38 MAPK activation triggered by UPEC. We postulate that the observed reduction in cytotoxicity and inflammation in AKB-4924 treated cells is due to partial blockade of UPEC-mediated p38 MAPK activation. Reduced inflammation in AKB-4924-treated bladders was reflected in significant reduction of proinflammatory cytokines and chemokines including IL-1β, IL-6, IL-8 (CXCL8) and KC (CXCL1), and diminished neutrophil recruitment. Our histology studies further indicate that AKB-4924 treatment reduces level of epithelial hyperplasia in the crypt of bladder, which reflects reduced chronic inflammation in the bladder [18,52,53]. These changes are likely reflective of the reduced bacterial burden. Our findings share similarities with recent studies in colitis and corneal infection models, where HIF-1α ultimately acted to suppress inflammation in the affected tissues. In the first study, Keely *et al*. showed up-regulation by AKB-4924 suppressed gastrointestinal tract inflammation in mice with chemically-induced colitis, with reduced IL-6, IL-1β and TNF-α production in both colons and blood sera [30]. The second study, silencing of HIF-1α transcription in the cornea led to greater bacterial burden, increased cytokine production and more MPO activity reflective of neutrophil infiltration [23]. Whereas intact pro-inflammatory signaling and neutrophil recruitment are critical for innate defense against bacterial pathogens, exaggerated levels of cytokines and recruited leukocytes can lead to persistent inflammation and recurrent injury to bladder epithelium, which could ultimately risk permanent renal scarring and functional impairment [17,18,68,69]. Thus, for the benefit of the host, it is important that the immune response remains tightly regulated and restores to homeostasis following infection.

UPEC attach to the surface of uroepithelial layers, where they rapidly colonize and invade epithelial cells, and can reside within these cells in vesicles or by escape into the cytoplasm to form IBC [12,42]. During the early phase of acute infections, UPEC induces a broad pro-inflammatory cytokine release in uroepithelial cells [6–9]. In this current study, we suspect that the reduction in inflammatory markers observed in the AKB-4924 treated uroepithelial cells and bladders is reflective of a diminished bacterial burden. Contributions of NO and AMPs to host defense against bladder and other infections have been well documented in the literature [10,23,55,56,58,70,71]. CRAMP-deficient animals experience increased susceptibility to UPEC UTI [58], and the robust killing effect of NO has also been clearly demonstrated in UTIs by various *E*. *coli* strains, including UPEC 1177, UPEC J96, ATCC25922, RK4353, and CM120 [56,72–74]. More recently, β-defensins have also been shown to play an antimicrobial role in protecting host against UTIs [75,76]. We found a significant difference in bacterial recovery between iNOS+/- and iNOS-/- mice 24 h post-infection. Although one study observed no overall difference in *E*. *coli* strain 1177 colonization between WT and iNOS-/- mice over a course of 7 d, they showed a 3-log fold increase in the bladders of iNOS-/- mice compared to WT mice 6h post-infection [56]. The discrepancy between our studies could also be due to different *E*. *coli* strains used, since NO susceptibility can vary depending on bacteria’s ability to detoxify NO [73,77], and could also due to different infection dosage, since higher dosage of infection does not necessarily enhance the infection severity (a lower infection dosage was used in our study). In this study we found AKB-4924 significantly boosted levels of NO, cathelicidins (human LL-37 and mouse CRAMP) and hBD2 in both UPEC infected human uroepithelial cells and murine bladders, coincides with studies that showed HIF-1α positively regulates NO and AMPs to support clearance of bacterial infections in skin keratinocytes and leukocytes [24,25,31,78,79]; thus we suggest that HIF-1α serves a similar role in uroepithelial cells. Future studies examining samples from human UTI patients will be required to precisely ascertain the antimicrobial role of NO and its relevance to human disease, since sometimes NO responses could be exaggerated in mice. Together, our results introduced HIF-1α as a key regulator of antimicrobial effectors providing host protection against UTI.

In summary, we have uncovered an interplay that exists between HIF-1α and the host innate immune response in the urinary tract. By depositing HIF-1α boosting agent directly into the bladder through a catheter, we could prevent infections and limit the risk of bladder and renal damage caused by acute inflammation. Since there are no known effective prophylactic agents available (other than antibiotics) to prevent UTIs [80], this application could serve as a prophylaxis to benefit certain high risk UTI patients, although administration of AKB-4924 to the urinary tract in an ascending fashion would likely be restricted to patients with indwelling catheters or those receiving daily catheterization. Therefore, a future goal of HIF boosting drugs would be to develop oral formulations that could effectively distribute into the urinary tract to reach wider patient populations.

HIF-1α activation occurs as a result of loss of vHL in certain forms of renal cell carcinoma, and HIF-1α controls VEGF and other angiogenic factors that are essential in support of tumor growth, which some considered potential risks of HIF-1 boosting therapy, however recent data indicates that activation of HIF-1 does not increase intestinal tumorigenesis [81]. Moreover, thorough Food and Drug Administration review has allayed these concerns, and allowed a number prolyl-hydroxlase inhibitor (HIF-1 boosting) drugs to enter human clinical trials for chronic administration in anemia patients, including FG-4592 (FibroGen, Astellas and AstraZeneca, ClinicalTrials.gov #NCT01887600, Phase III), AKB-6548 (Akebia Therapeutics, #NCT01235936, Phase II), and GSK1278863 (Glaxo-SmithKline, #NCT01977573, Phase II) [82]. We conclude that AKB-4924 and similar HIF-1α boosting agents could merit further exploration as novel therapeutic tools in the prevention and treatment UPEC-related UTIs.

## Materials and Methods

### Ethics statement

This study was carried out in strict accordance with the recommendations in the Guide for the Care and Use of Laboratory Animals of the National Institutes of Health. The protocol was approved by the Institutional Animal Care and Use Committee of the University of California, San Diego (Animal Welfare Assurance Number: A3033-01). All efforts were made to minimize suffering of animals employed in this study.

### Animals

WT C57BL/6 mice aged 6–7 weeks were purchased from Jackson laboratory. HIF-1α knockout targeted to keratinocyte inactivation of HIF-1α (*Hif1α* ^*flox/flox*^/K14-Cre^+^) and WT littermates from the same breeding pair were used at 6–7 weeks [24]; iNOS^+/-^ (heterozygous) or iNOS^-/-^ (null) C57BL/6 mice (Jackson Laboratory, strain B6.129P2-NOS2^tm1Lau^/J) littermates from the same breeding pairs were used at 7–10 weeks [83]. Mice were handled by approved protocols of the UCSD Animal Care Committee.

### Bacterial strain, cells, media and growth conditions

Wild-type uropathogenic *E*. *coli* (UPEC) strain CFT073 (O6:K2:H1; ATCC 700928) and UTI89 were grown for at least 20 h in standing culture to stationary phase at 37°C in Luria-Bertani (LB) broth prior to infection. Human uroepithelial cell line 5637 (ATCC# HTB-9) were cultured in RPMI-1640 (Invitrogen) media supplemented with 10% heat-inactivated fetal bovine serum (FBS) at 37°C in humidified air with 5% CO_2_.

### AKB-4924

AKB-4924 (Aerpio Therapeutics, Cincinnati, OH) was manufactured as previously described [29]. For *in vivo* experiments, each mouse was treated transurethrally with 0.2 mg of AKB-4924 prepared in 40% 2-hydroxylpropyl-β-cyclodextran in 50 mM aqueous citrate buffer at pH 5. For *in vitro* experiments, cells in RPMI-1640 supplemented with 10% FBS were treated for 2 h with 20 M of AKB-4924 resuspended in DMSO pH = 4.2–4.4 unless otherwise stated.

### *In vitro* UPEC infection of cultured bladder epithelial cells

Human uroepithelial cells 5637 were seeded at ~2 x 10^5^ cells/mL in 24-well plates a day prior to AKB-4924 pre-treatment. Confluent 5637 cells were treated with 0.4% of DMSO (control) or 20 μM AKB-4924 prepared in DMSO (Aerpio Therapeutics) for 2–4 h and were infected with UPEC from a fresh overnight standing culture (OD ~0.5) at a multiplicity of infection (MOI) ~20. Plates were centrifuged at 650 x *g* for 5 min to facilitate bacterial contact with the host cell monolayer. The bacteria were then allowed to establish attachment on monolayers for 2 h in the presence of AKB-4924 or DMSO. After 2 h infection, cells were washed 3x with PBS lysed by adding 100 μL of 0.05% trypin (1 min) followed by 900 μL of 0.025% triton X-100 in PBS to assess total surviving bacteria by CFU enumeration. To assess the level of internalized bacteria, cells were washed with fresh media 2 h post-infection and treated with 100 μg/mL gentamicin to kill extracellular bacteria for 2 h before CFU recovery as above [39].

### Mouse urinary tract infection model

An established mouse UTI protocol was used as previously described [49]. Urine is voided from the bladder of all animals prior to transurethral treatment with 2 mg/ml of AKB-4924 in 100 μL (administered in ~4 sec) for 1 h followed by infection with 50 μL of UPEC CFT073 (administered in ~2–3 sec) at ~2–5 x 10^8^ colony forming unit (CFU) per mouse or UTI89 at ~6 x 10^7^ CFU per mouse. Alternatively, mice were treated with AKB-4924 6h post-infection. Transurethral infection was achieved by inserting an UV-sterilized polyethylene tube (inner dimension 0.28 mm, outer dimension 0.61 mm, Catalogue number 598321 Harvard Apparatus) attached to a 30g hypodermic needle into the urethra. At ~20 h post-infection, urine samples were collected from each mouse. Bladders and both kidneys were removed and homogenized using MagNa Lyser (Roche) for CFU enumeration. For an *ex vivo* gentamicin protection assay, bladders were removed from mice ~18 h post-infection and washed three times with PBS followed by treatment with 100 μg/mL of gentamicin for 2 h to kill extracellular bacteria. Bladders were washed then three times, homogenization in 1 mL PBS + 0.025% triton, and serially diluted for CFU enumeration. All infection is performed using UPEC CFT073 unless otherwise stated.

### Live/dead staining

Confluent 5637 monolayers were grown on sterile no. 1 round coverslips (Thermo Scientific) in 24-well or 6-well plates. Cells were infected with WT UPEC at a MOI of 5–20 for 2 h. Cells were washed with PBS and treated with the viability assay mixture from the LIVE/DEAD Viability/Cytotoxicity Kit for mammalian cells (Molecular Probes, Invitrogen) for 30 min at 37°C, then mounted on glass slides for visualization and imaging using an Olympus BX51 fluorescent microscope fitted with appropriate filters as described previously [39].

### Histology and immunohistochemistry

Tissues for H&E staining were submerged in formalin overnight at 4°C and transferred to the Histopathology Core facility for processing. For Immunohistochemistry, immediately after euthanasia, the entire bladder was removed from the mouse and submerged in pre-warmed 3% paraformaldehyde for 3 h. Tissues were washed 3 times, 10 min each in 10 mL PBS and immediately frozen down in OCT compound with liquid nitrogen for 5 μM sectioning by the UCSD Mouse Histopathology Core facility (N. Varki, Director). Tissue sections were immediately submerged into 0.2% Triton X-100 in PBS for 5 min and washed extensively with PBS before treatment with 5% normal goat serum (NGS) in TPBS/BSA for 20 min at room temperature. Tissues were incubated with rabbit anti-cathelicidin (Catalog No. NB100-98689, Novus Biological) at 1:200 dilution, rabbit anti-MPO polyclonal antibody (Catalogue No. RB373A, Thermo Scientific) at 1:200 dilution or rabbit (DA1E) IgG monoclonal isotype control antibody (Catalog No. 3900, Cell Signaling Technology) at 1:200 in 1% NGS in TPBS/BSA overnight at 4°C. On the next day, coverslips were washed with TPBS/BSA 3 times and incubated with a goat anti-rabbit Alexa 594 antibody (Molecular probes) for 1 h at 37°C before they were washed and mounted with ProLong Gold anti-fade reagent with DAPI (Catalogue No. P-36931 Molecular Probes). Cells were visualized using an Olympus BX51 fluorescent microscope fitted with appropriate filters.

### Myeloperoxidase and nitrite assays

The level of neutrophil migration into mouse bladders was determined by myeloperoxidase (MPO) assay. MPO activity released in bladder homogenates was quantified by the MPO colorimetric activity assay (Catalogue No. MAK068, Sigma Aldrich) as previously described [33]. Greiss reagent (Catalog No. G2930, Promega) was used to detect nitrite production in 5637 cells according to the manufacturer’s protocol.

### Nuclear extraction

Confluent 5637 cells in T75 flask were washed with PBS once before treated with 20 mM 4924 or DMSO in 25 mL fresh media for 4 h at 37°C. Cells were harvested by 5 min treatment with 0.05% tryspin and 15 mL chilled media followed by centrifugation at 400 x *g* for 10 min at 4°C. Pellets were re-suspended in 1 mL PBS at 4°C and centrifuged for 5 min. The supernatant was collected for subsequent nuclear extraction using the NE-PER Nuclear and cytoplasmic extraction kit following manufacturer’s protocol (Catalogue No. 78833, Thermo Scientific).

### Western blotting

Whole cell lysates from 5637 were prepared for western blot as previously described [39]. The following antibodies were used: rabbit anti-HIF-1α antibody, rabbit anti-HIF-2α (NB100-122) (Novus Biological), mouse anti-P84 antibody (Catalog No. GTX70220) (GeneTex), rabbit anti-paxillin, rabbit anti-phospho-p65 (Ser536) monoclonal antibody (Catalogue No. 3033) and rabbit anti-phospho-p38 (Thr180/Tyr182) polyclonal antibody (Catalogue No. 92122) (Cell Signaling Technologies). Bladder homogenates were prepared by lysing a bladder in 500 μL RIPA lysis buffer with beads using the MegaLyser (Roche). Cathelicidin detection was made using the same anti-cathelicidin antibody as immunolocalization. Anti-mouse β-actin monoclonal antibody clone AC-74 (Catalogue No. A5316, Sigma-Aldrich) was used as a loading control. Band intensity was measured using Image J software.

### Cytokine quantification

Concentrations of cytokines were measured in supernatant from infected 5637 cells (2h post-infection) or homogenized bladder or kidneys 1 d post infection in mice (n > 3). Levels of mouse or human (5637 cells) cytokines IL-6, and IL-1β were detected using ELISA kits following the manufacturer’s protocol (R&D systems). Assays were performed in triplicates or quadruplicates for each experiment.

### Real-time quantitative PCR

For tissue cultures, 500 μL of TRIZOL reagent was added each well whereas for bladder and kidney isolates, 1 mL of TRIZOL was added. After RNA isolates were re-suspended in RNAase/DNase- free water and TURBO DNase (Ambion, Invitrogen) was added to eliminate potential DNase contamination in the RNA prep. To synthesize cDNA, approximately 100 ng of total RNA was used for iScript cDNA Synthesis kit (Bio-Rad). Approximately 1 ng of cDNA was used in triplicates or quadruplicates for real-time qPCR using KAPA SYBR qPCR 2x master mix (KAPA Biosystem, Catalog# KM4101). The reaction was performed using the Biorad CFX96 Real-time C1000 Thermocycler. Primers were used at a final concentration of 200nmol. Primer sequences used in this study are listed in S1 Table. Mouse β-2-microglobulin (β2M) and human β-actin were used as control house keeping genes. Relative transcript level was normalized to endogenous house keeping genes using the 2^-ΔΔCt^ method [84].

### Motility assays

Overnight cultures of UPEC CFT073 was grown in LB and inoculated on swimming LB plates with 0.25% of agar supplemented with vehicle or 0.2mg/mL AKB-4924 (used for *in vivo* study), 0.4% DMSO or 20 M AKB-4924 (used for *in vitro* study). Bacteria were grown at 37°C over 18 h and swimming diameter was measured (n = 3).

### Statistical analysis

All experiments were performed in triplicates or quadruplicate, and repeated in at least two independent experiments. All data are presented as mean and error represents S.E.M. (n > 3) from multiple independent experiments. Statistical analysis is performed using One-way ANOVA or Student’s unpaired two-tailed t-test (Graph Pad Prism, version 5.03). *P < 0.05, **P < 0.01 and ***P < 0.001 represent statistical significance; *P* > 0.05 or n.s. is non significant.

## Supporting Information

S1 Fig AKB-4924 decreases UPEC UTI89 infection in human uroepithelial cells. Bacterial counts from untreated (UT), 2 h DMSO or AKB-4924 treated 5637 cells followed by 2 h infection with UPEC UTI89 to measure total bacteria (left), or 2 h infection with additional 2 h gentamicin (100 μg/mL) treatment (right) to measure intracellular bacteria (n = 3 per group). Error bar = S.E.M., *P < 0.05, **P < 0.01, One way ANOVA followed by Tukey’s post-test. Results are representative experiment from two independent experiments.
(TIF)

S2 Fig Western blot of UPEC infected mouse bladders and controls for uninfected bladder epithelial cells. (A) UPEC CFT073 infected (vehicle or AKB-4924 treated) and untreated (UT) bladders that were homogenized in RIPA lysis buffer 24 h post-infection. (B) 5637 cells that are treated with 0.4% DMSO or 20 μM AKB-4924 (2 h) display identical protein expression profiles (C) AKB-4924 treated cells displayed elevated HIF-2α proteins compared to mock (DMSO treated cells). These are representative ςestern blots from two independent experiments.
(TIF)

S3 Fig UPEC growth controls. A) Real-time qPCR showing relative HIF-1 expression in uninfected or UPEC CFT073 infected bladders from C57BL/6 mice (24 h infection) (n = 3). (B) AKB-4924 does not influence UPEC growth in mouse urine, PBS or RPMI supplemented with 10% fetal bovine serum (FBS). Urine from ~10 different 6–12 week old C57BL/6 female mice were collected. Growth of UPEC CFT073 in the urine, PBS or 10% FBS + RPMI supplemented with vehicle or 0.2 mg of AKB-4924 was maintained at 37°C stationary culture and assayed for CFU recovery over time. (C) Swimming motility assay. UPEC were grown on 0.25% LB agar plate supplemented with 0.4% DMSO or 20uM AKB-4924 overnight. Swimming diameters were measured from triplicate samples (n = 3). Data shown as mean +/- S.E.M., *P < 0.05; *P* >0.05 = n.s (not significant), Student’s unpaired t-test. Results are pooled from three independent experiments.
(TIF)

S4 Fig Time course analysis of VEGF expression and bacterial burden in AKB-4924 treated mice challenged with UTIs. C57BL/6 mice were treated transurethrally with vehicle or 0.2 mg of AKB-4924 for 1 h and challenged with UPEC CFT073 for 0 h, 4 h, 8 h, and 20 h. Bladders were harvested and homogenized for ELISA assay and CFU enumeration. (A) ELISA quantifies VEGF protein in bladder homogenates. Left panel (uninfected, t = 0), right panel 4, 8, 20 h (n = 8) (B) CFU recovery from bladders (n>6). Closed circle = vehicle (placebo control), open circle = 4924. Shown as mean +/- S.E.M., *P < 0.05; *P* > 0.05 = n.s (not significant), Student’s unpaired t-test.
(TIF)

S5 Fig Histology of bladder sections from uninfected and UPEC-infected mice. Representative images of C57BL/6 mice left untreated or treated with vehicle or 0.2 mg of AKB-4924 for 1 h prior to infection with UPEC CFT073 for 18h. Mice were sacrificed and bladders were sterilely removed for H&E staining. Vehicle-treated mice with UPEC infection suffer severe hyperplasia and inflammation, as evident in thinning of the superficial epithelial layer and increases in crypt length compared to AKB-4924-treated and uninfected mice. Arrows indicate intact superficial uroepithelial layers in some regions of the tissue; asterisk indicates luminal region. (n = 2 uninfected; n = 4 infected, from 3 independent experiments). Scale bar = 200 μM.
(TIF)

S6 Fig Immunolocalization microscopy controls. (A) Normal rabbit IgG control. Representative image of UTI89 infected bladder (vehicle treated) stained with anti-rabbit IgG at 1:200 dilution followed by detection with Alexa 594. (B) MPO localization of uninfected bladder. Representative image of untreated bladder stained with anti-MPO antibody as described in materials and method. DAPI = nuclei staining. Scale bar = 50 μM.
(TIF)

S7 Fig Effect of AKB-4924 on wild-type or iNOS knockdown human uroepithelial cells. (**A**) Real-time qPCR shows 2h AKB-4924 treatment at 20uM increases iNOS mRNA level in human uroepithelial cells (n = 8). (**B**) Bacteria were incubated for 2 h in filter-sterilized supernatant isolated from uninfected or UPEC infected (2h) cells treated with DMSO or 4924 (20 μm). Bacterial recovery = % recovered from UPEC grown in infected vs. uninfected cell supernatant (n = 5). Shown as mean +/- S.E.M., *P < 0.05, Student’s unpaired t-test.
(TIF)

S1 Table Primers used for real-time quantitative PCR. (DOCX)
