# Supplementary figures and images for: Role of Hypoxia Inducible Factor-1α (HIF-1α) in Innate Defense against Uropathogenic Escherichia coli Infection
Source: PLoS Pathog. 2015 Apr 30;11(4):e1004818. doi: 10.1371/journal.ppat.1004818 (PMC4415805; doi:10.1371/journal.ppat.1004818)

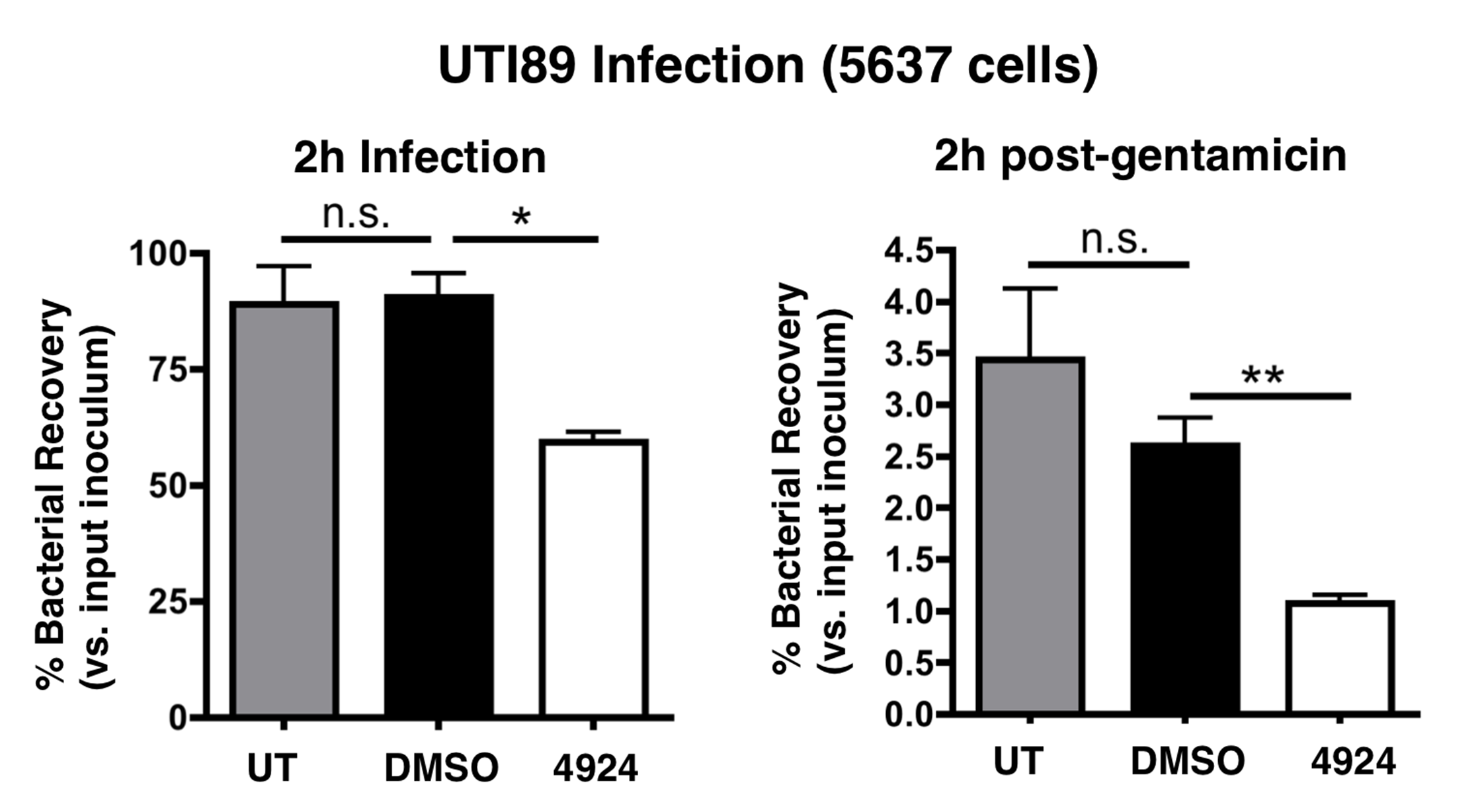

Supplement: S1 Fig — Bacterial counts from untreated (UT), 2 h DMSO or AKB-4924 treated 5637 cells followed by 2 h infection with UPEC UTI89 to measure total bacteria (left), or 2 h infection with additional 2 h gentamicin (100 μg/mL) treatment (right) to measure intracellular bacteria (n = 3 per group). Error bar = S.E.M., *P < 0.05, **P < 0.01, One way ANOVA followed by Tukey’s post-test. Results are representative experiment from two independent experiments. (TIF) [file ppat.1004818.s001.tif]

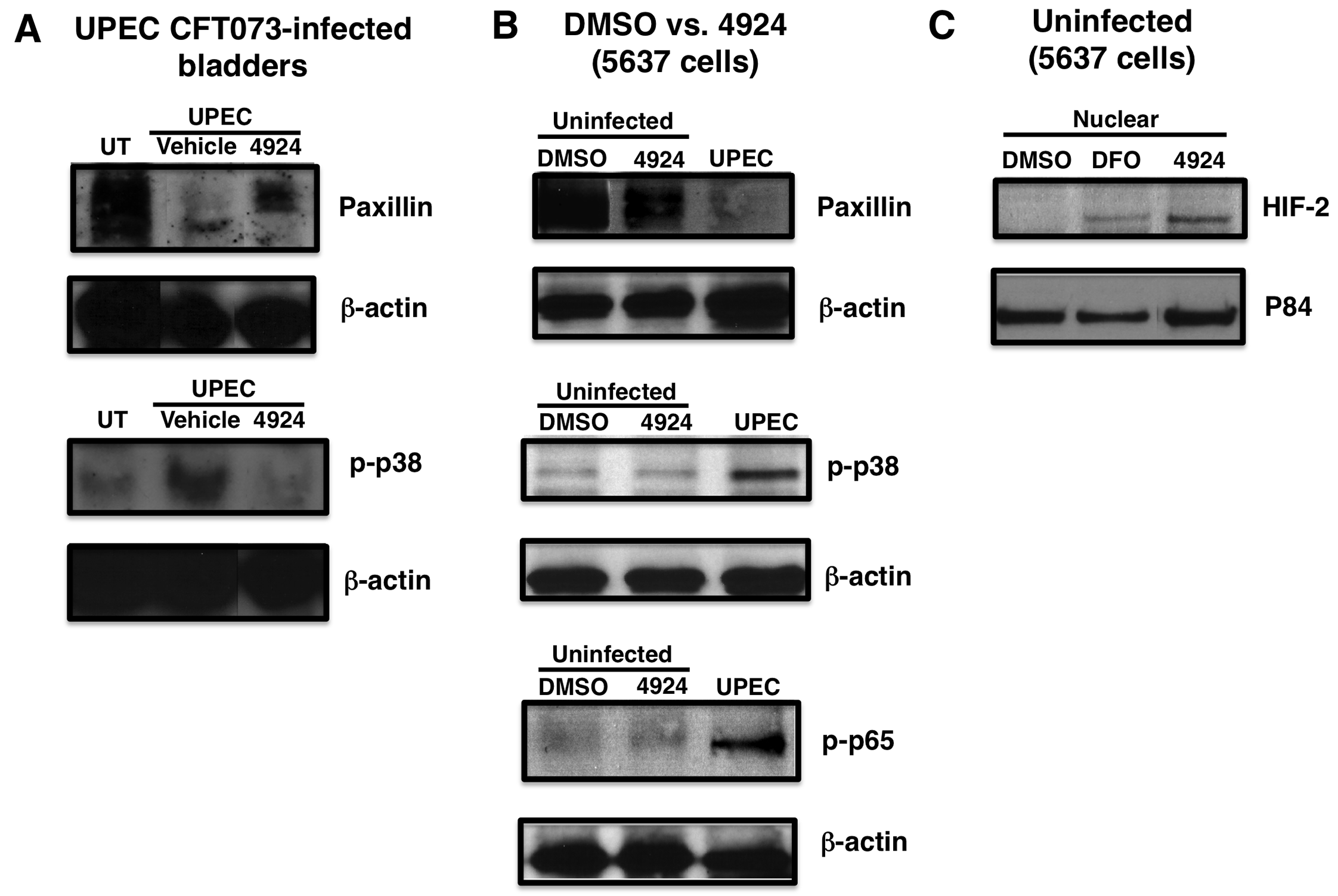

Supplement: S2 Fig — (A) UPEC CFT073 infected (vehicle or AKB-4924 treated) and untreated (UT) bladders that were homogenized in RIPA lysis buffer 24 h post-infection. (B) 5637 cells that are treated with 0.4% DMSO or 20 μM AKB-4924 (2 h) display identical protein expression profiles (C) AKB-4924 treated cells displayed elevated HIF-2α proteins compared to mock (DMSO treated cells). These are representative ςestern blots from two independent experiments. (TIF) [file ppat.1004818.s002.tif]

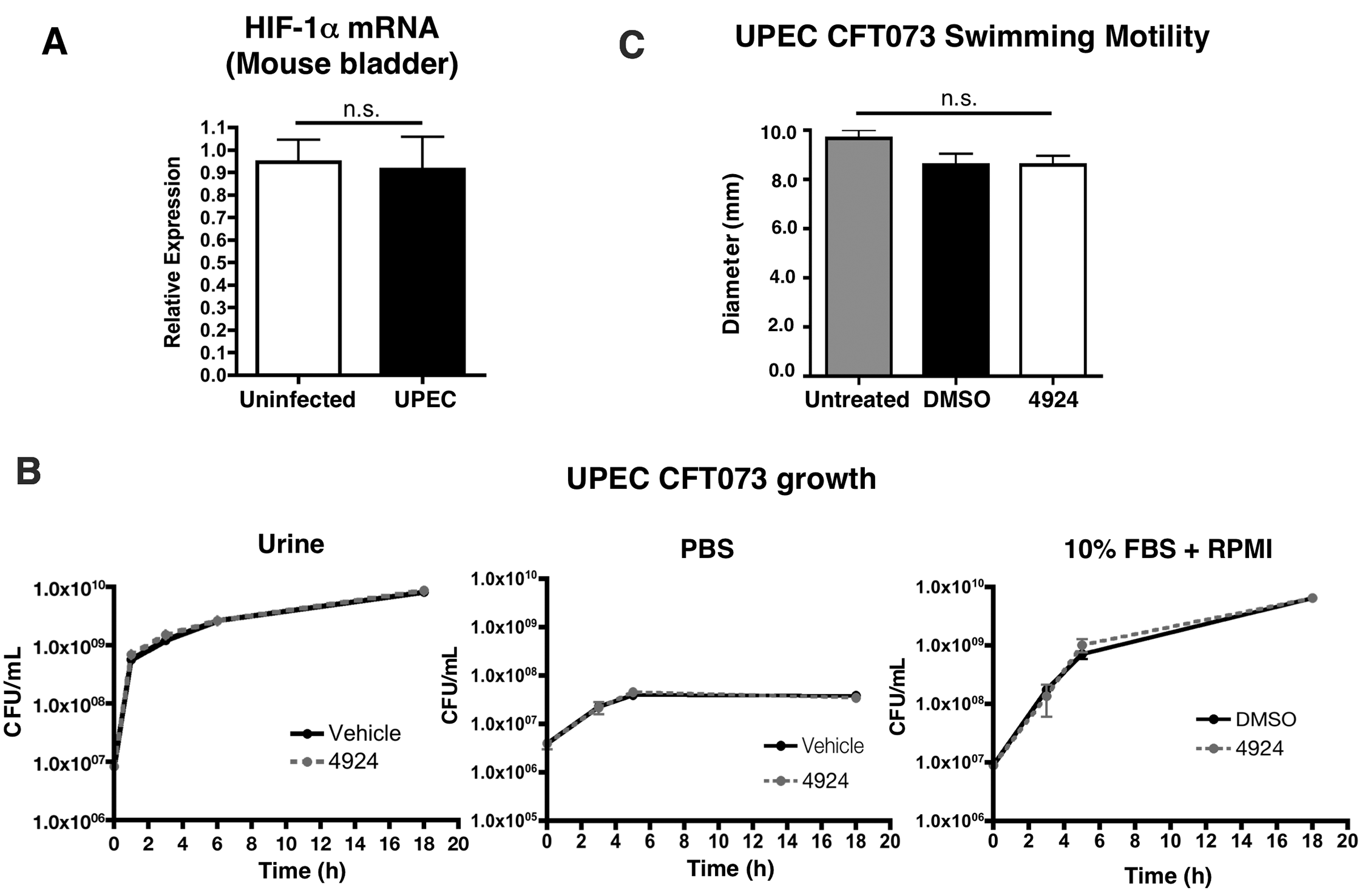

Supplement: S3 Fig — A) Real-time qPCR showing relative HIF-1 expression in uninfected or UPEC CFT073 infected bladders from C57BL/6 mice (24 h infection) (n = 3). (B) AKB-4924 does not influence UPEC growth in mouse urine, PBS or RPMI supplemented with 10% fetal bovine serum (FBS). Urine from ~10 different 6–12 week old C57BL/6 female mice were collected. Growth of UPEC CFT073 in the urine, PBS or 10% FBS + RPMI supplemented with vehicle or 0.2 mg of AKB-4924 was maintained at 37°C stationary culture and assayed for CFU recovery over time. (C) Swimming motility assay. UPEC were grown on 0.25% LB agar plate supplemented with 0.4% DMSO or 20uM AKB-4924 overnight. Swimming diameters were measured from triplicate samples (n = 3). Data shown as mean +/- S.E.M., *P < 0.05; P >0.05 = n.s (not significant), Student’s unpaired t-test. Results are pooled from three independent experiments. (TIF) [file ppat.1004818.s003.tif]

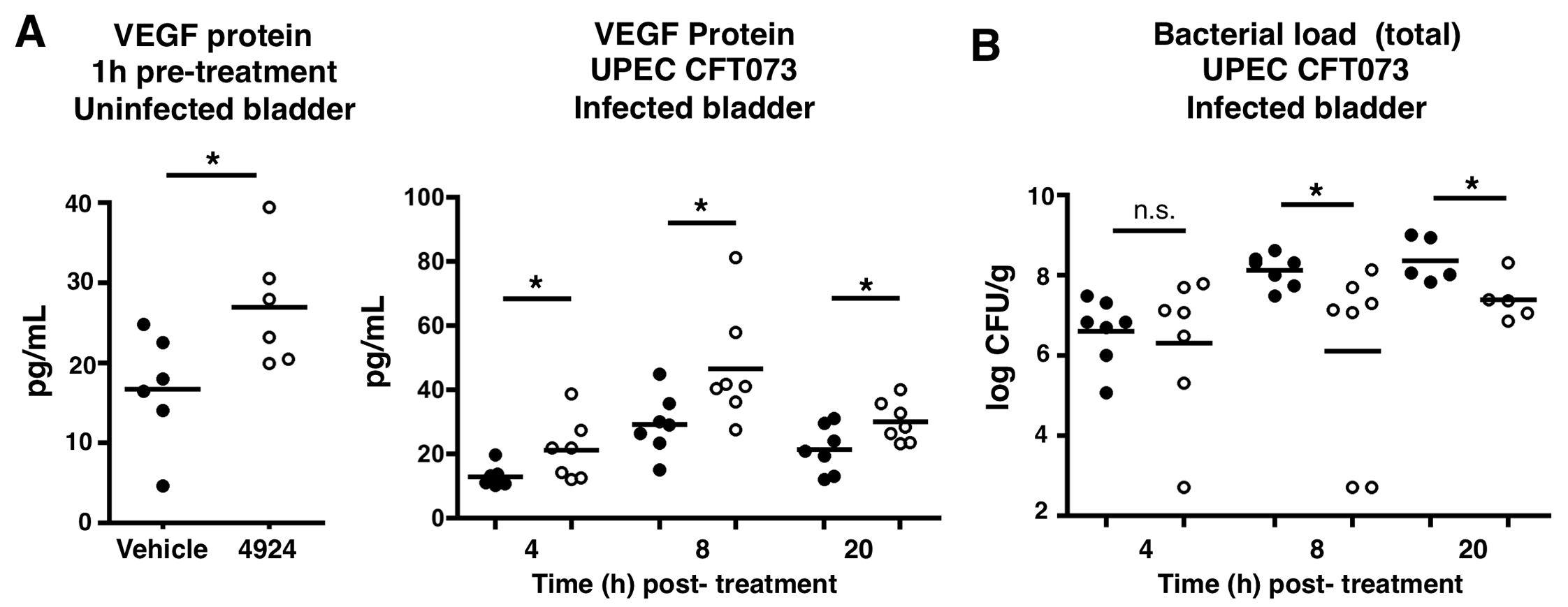

Supplement: S4 Fig — C57BL/6 mice were treated transurethrally with vehicle or 0.2 mg of AKB-4924 for 1 h and challenged with UPEC CFT073 for 0 h, 4 h, 8 h, and 20 h. Bladders were harvested and homogenized for ELISA assay and CFU enumeration. (A) ELISA quantifies VEGF protein in bladder homogenates. Left panel (uninfected, t = 0), right panel 4, 8, 20 h (n = 8) (B) CFU recovery from bladders (n>6). Closed circle = vehicle (placebo control), open circle = 4924. Shown as mean +/- S.E.M., *P < 0.05; P > 0.05 = n.s (not significant), Student’s unpaired t-test. (TIF) [file ppat.1004818.s004.tif]

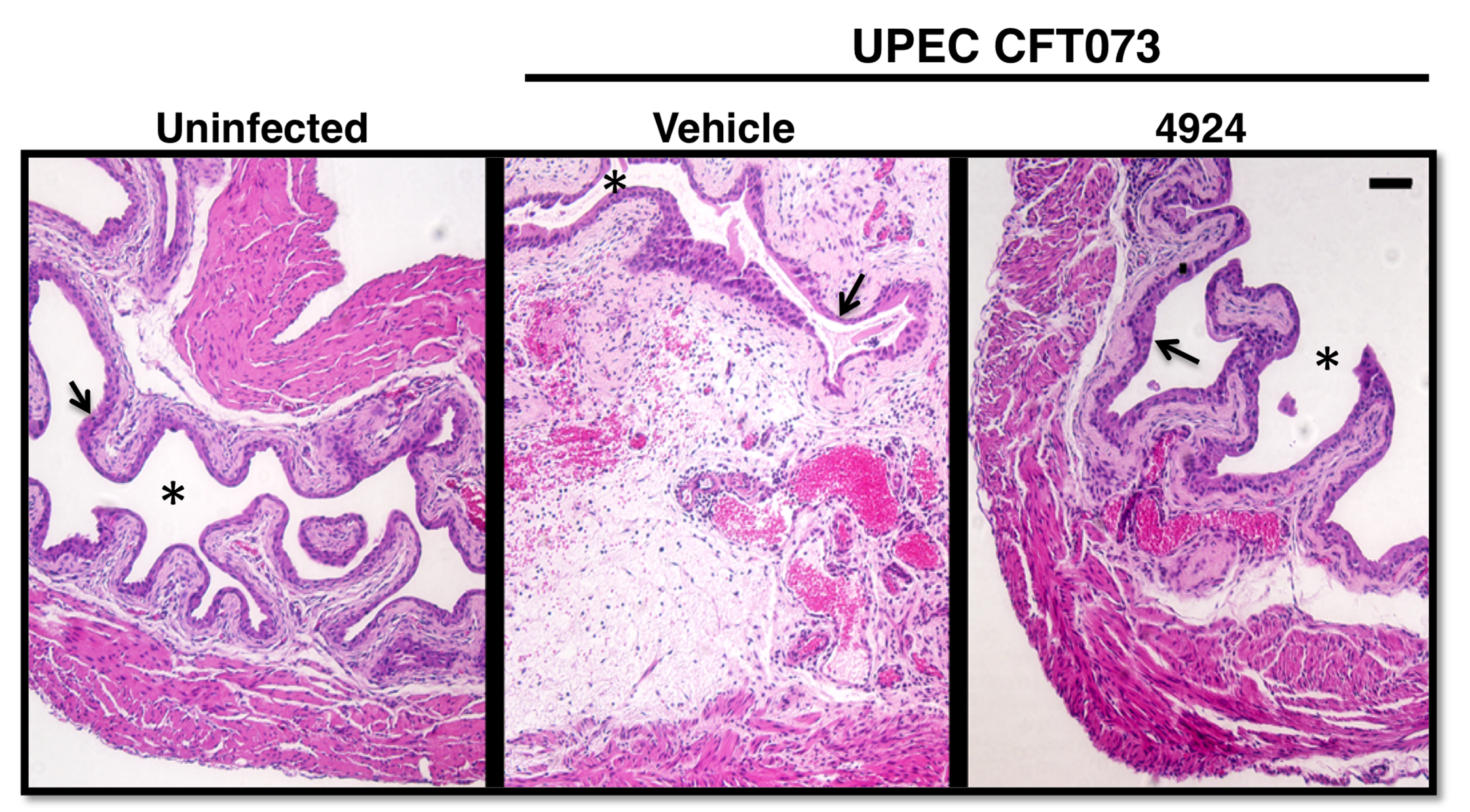

Supplement: S5 Fig — Representative images of C57BL/6 mice left untreated or treated with vehicle or 0.2 mg of AKB-4924 for 1 h prior to infection with UPEC CFT073 for 18h. Mice were sacrificed and bladders were sterilely removed for H&E staining. Vehicle-treated mice with UPEC infection suffer severe hyperplasia and inflammation, as evident in thinning of the superficial epithelial layer and increases in crypt length compared to AKB-4924-treated and uninfected mice. Arrows indicate intact superficial uroepithelial layers in some regions of the tissue; asterisk indicates luminal region. (n = 2 uninfected; n = 4 infected, from 3 independent experiments). Scale bar = 200 μM. (TIF) [file ppat.1004818.s005.tif]

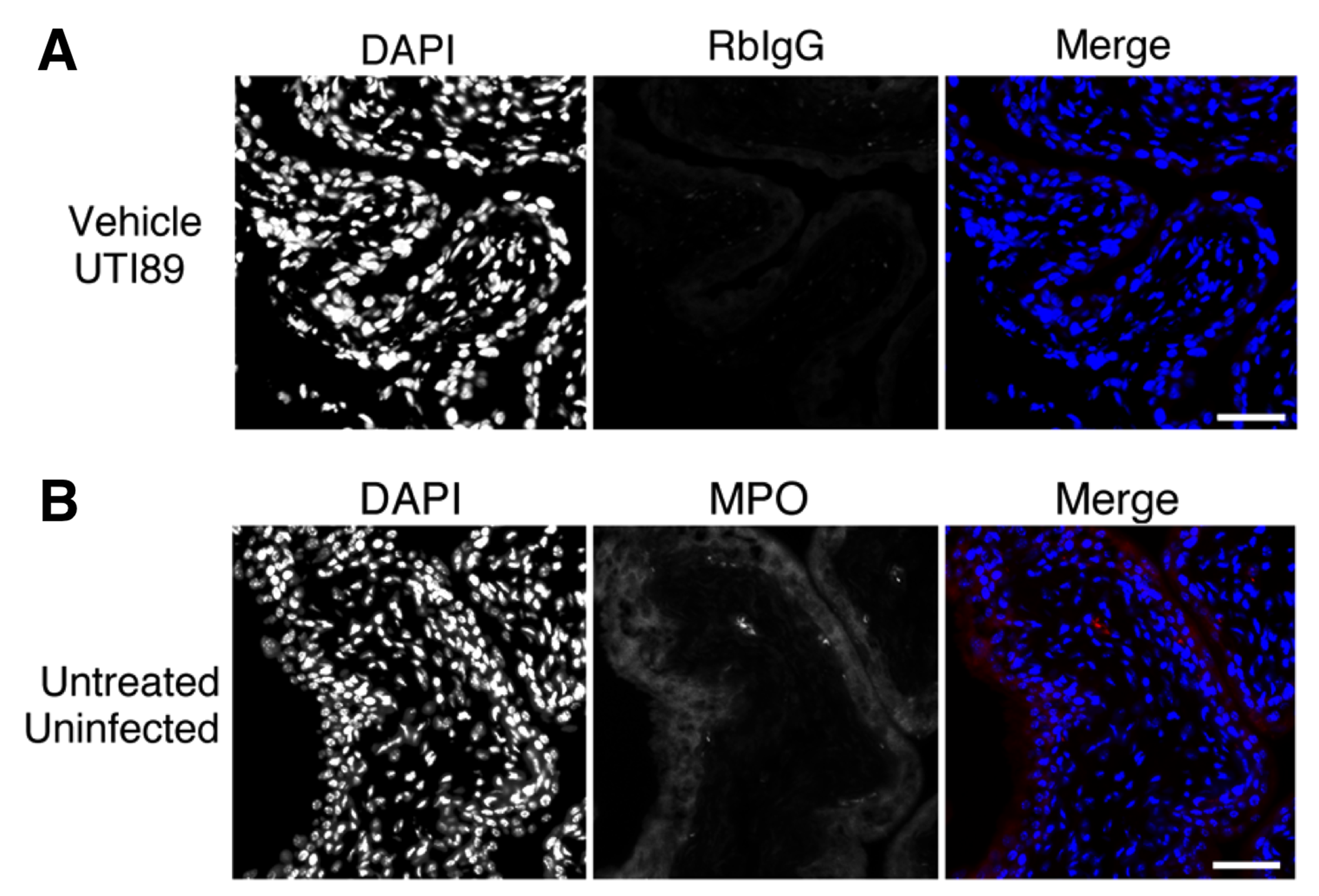

Supplement: S6 Fig — (A) Normal rabbit IgG control. Representative image of UTI89 infected bladder (vehicle treated) stained with anti-rabbit IgG at 1:200 dilution followed by detection with Alexa 594. (B) MPO localization of uninfected bladder. Representative image of untreated bladder stained with anti-MPO antibody as described in materials and method. DAPI = nuclei staining. Scale bar = 50 μM. (TIF) [file ppat.1004818.s006.tif]

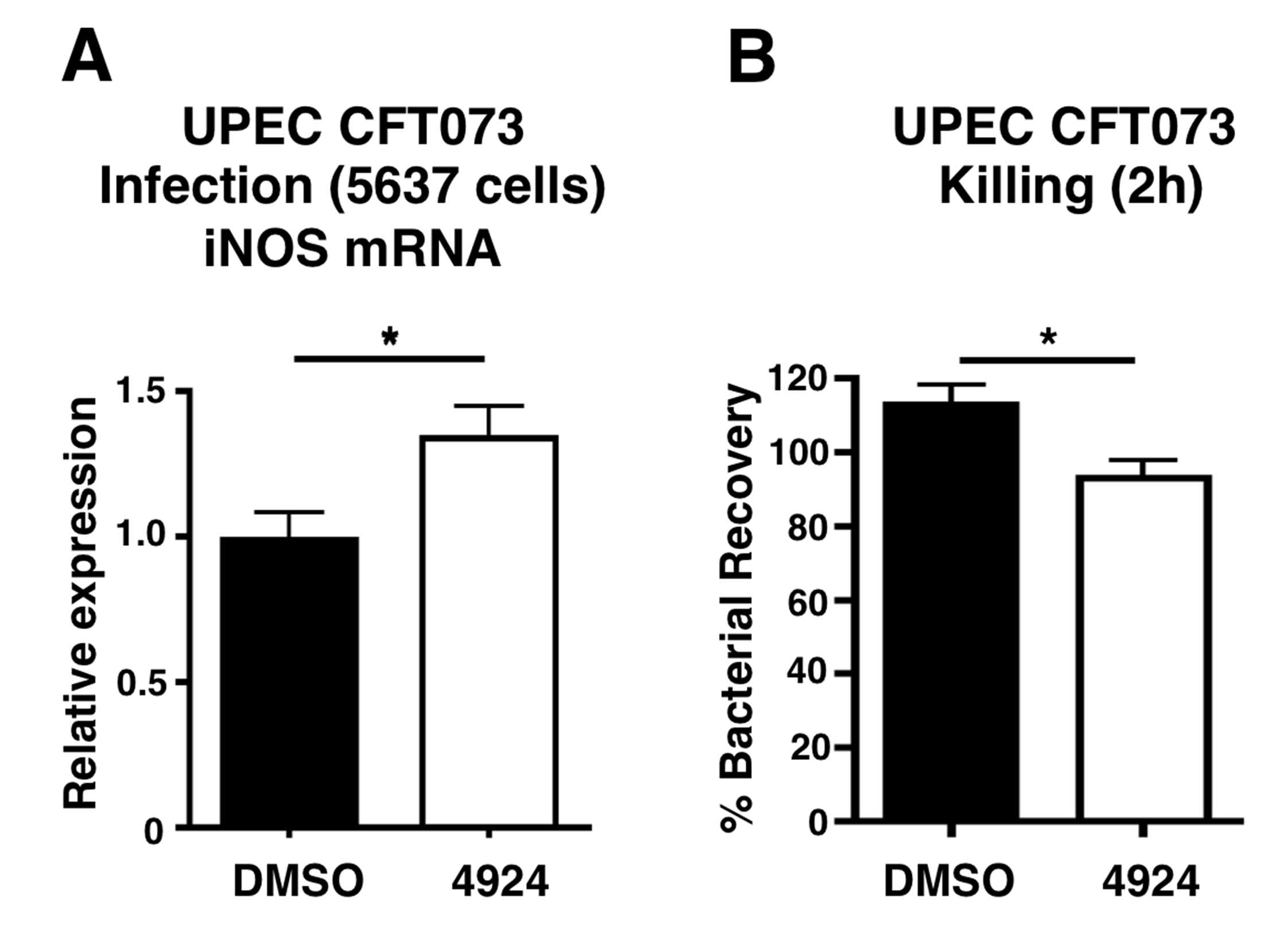

Supplement: S7 Fig — (A) Real-time qPCR shows 2h AKB-4924 treatment at 20uM increases iNOS mRNA level in human uroepithelial cells (n = 8). (B) Bacteria were incubated for 2 h in filter-sterilized supernatant isolated from uninfected or UPEC infected (2h) cells treated with DMSO or 4924 (20 μm). Bacterial recovery = % recovered from UPEC grown in infected vs. uninfected cell supernatant (n = 5). Shown as mean +/- S.E.M., *P < 0.05, Student’s unpaired t-test. (TIF) [file ppat.1004818.s007.tif]
