# Supplementary material for: Role of Hypoxia Inducible Factor-1α (HIF-1α) in Innate Defense against Uropathogenic Escherichia coli Infection
Source: PLoS Pathog. 2015 Apr 30;11(4):e1004818. doi: 10.1371/journal.ppat.1004818 (PMC4415805; doi:10.1371/journal.ppat.1004818)
Supplement: S1 Table — (DOCX) [file ppat.1004818.s008.docx]

**Supplemental Table S1. Primers used for real-time quantitative PCR.**

| **Target** | **Sequence (5’-3’)** | **Ref.** |
| --- | --- | --- |
| human LL-37 | F 5’-ACC CAG CAG GGC AAA TCT-3’  R 5’-GAA GGA CGG GCT GGT GAA-3’ | [[1](#_ENREF_1)] |
| human β-defensin 2 (hBD2) | F 5’-CCC TTT CTG AAT CCG C-3’  R 5’-GAG GGT TT GTA TCT CCT-3 | [[2](#_ENREF_2)] |
| human β-actin | F 5’-AAG AGA GGC ATC CTC ACC CT-3’  R 5’-TAC ATC GCT GGG GTG TTG-3’ | [[3](#_ENREF_3)] |
| human HIF-1α | F 5’-GCT GGC CCC AGC CGC TGG AG- 3’  R 5’-GAG TGC AGG GTC AGC ACT AC-3’ | [[4](#_ENREF_4)] |
| human iNOS | F 5’-CAG CGG GTG ACT TTC CAA -3’  R 5’- AGG CAA GAT TTG GAC CTG CA -3’ | [[5](#_ENREF_5)] |
| human VEGF | F 5’-CTT CCT ACA GCA CAA CAA AT-3’  R 5’ GTC TTG CTC TAT CTT TCT TTG G-3’ | [[7](#_ENREF_7)] |
| mouse HIF1α | F 5’-GAA ACG ACC ACT GCT AAG GCA-3’  R 5’-GGC AGA CAG GTT AAG GCT CCT-3’ | [[6](#_ENREF_6)] |
| mouse VEGFA | F 5’-ACT GGA CCC TGG CTT TAC TG-3’  R 5’-TCT GCT CTC CTT CTG TCG TG-3’ | [[7](#_ENREF_7)] |
| CRAMP | F 5’-AAT TTT CTT GAA CCG AAA GGG C-3’  R 5’-TGT TTT CTC TCA GAT CCT TGG GAG C-3’ | [[8](#_ENREF_8)] |
| mouse β-defensin2  (mBD2) | F 5’-TCT CTG CTC TCT GCT GCT GAT ATG C-3’  R 5’-AGG ACA AAT GGC TCT GAC ACA GTA CC-3’ | [[6](#_ENREF_6)] |
| mouse iNOS | F 5’-GTT CTC AGC CCA ACA ATA CAA GA-3’  F 5’-GTG GAC GGG TCG ATG TCA C-3’ | [[9](#_ENREF_9)] |
| Mouse β2-microglobulin (B2M) | F 5’-CCC CAC TGA GAC TGA TAC ATA CG-3’  R 5’-CGA TCC CAG TAG ACG GTC TTG-3’ | [[10](#_ENREF_10)] |

1. Schauber J, Svanholm C, Termen S, Iffland K, Menzel T, et al. (2003) Expression of the cathelicidin LL-37 is modulated by short chain fatty acids in colonocytes: relevance of signalling pathways. Gut 52: 735-741.

2. Wehkamp J, Harder J, Wehkamp K, Wehkamp-von Meissner B, Schlee M, et al. (2004) NF‑κB- and AP-1-mediated induction of human β-defensin-2 in intestinal epithelial cells by *Escherichia coli* Nissle 1917: a novel effect of a probiotic bacterium. Infect Immun 72: 5750-5758.

3. Bhandari T, Olson J, Johnson RS, Nizet V (2013) HIF-1α influences myeloid cell antigen presentation and response to subcutaneous OVA vaccination. J Mol Med (Berl) 91: 1199-1205.

4. Frede S, Stockmann C, Winning S, Freitag P, Fandrey J (2009) Hypoxia-inducible factor (HIF)-1α accumulation and HIF target gene expression are impaired after induction of endotoxin tolerance. J Immunol 182: 6470-6476.

5. Guo Z, Shao L, Zheng L, Du Q, Li P, et al. (2012) miRNA-939 regulates human inducible nitric oxide synthase posttranscriptional gene expression in human hepatocytes. Proc Natl Acad Sci U S A 109: 5826-5831.

6. Berger EA, McClellan SA, Vistisen KS, Hazlett LD (2013) HIF-1α is essential for effective PMN bacterial killing, antimicrobial peptide production and apoptosis in *Pseudomonas aeruginosa* keratitis. PLoS Pathog 9: e1003457.

7. Kase S, He S, Sonoda S, Kitamura M, Spee C, et al. (2010) αB-crystallin regulation of angiogenesis by modulation of VEGF. Blood 115: 3398-3406.

8. Li D, Beisswenger C, Herr C, Schmid RM, Gallo RL, et al. (2013) Expression of the antimicrobial peptide cathelicidin in myeloid cells is required for lung tumor growth. Oncogene.

9. Pindado J, Balsinde J, Balboa MA (2007) TLR3-dependent induction of nitric oxide synthase in RAW 264.7 macrophage-like cells via a cytosolic phospholipase A2/cyclooxygenase-2 pathway. J Immunol 179: 4821-4828.

10. Wree A, Eguchi A, McGeough MD, Pena CA, Johnson CD, et al. (2014) NLRP3 inflammasome activation results in hepatocyte pyroptosis, liver inflammation, and fibrosis in mice. Hepatology. 59: 898-910.
